# Age-related elevation of O-GlcNAc causes meiotic arrest in male mice

**DOI:** 10.1038/s41420-023-01433-x

**Published:** 2023-05-15

**Authors:** Zhang Qian, Chuwei Li, Shanmeizi Zhao, Hong Zhang, Rujun Ma, Xie Ge, Jun Jing, Li Chen, Jinzhao Ma, Yang Yang, Lu Zheng, Kemei Zhang, Zhaowanyue He, Mengqi Xue, Ying Lin, Kadiliya Jueraitetibaike, Yuming Feng, Chun Cao, Ting Tang, Shanshan Sun, Hui Teng, Wei Zhao, Bing Yao

**Affiliations:** 1 Center of Reproductive Medicine, Affiliated Jinling Hospital, Medical School, Nanjing University, Nanjing, 210002 Jiangsu China; 2 Jiangsu Key Laboratory for Molecular and Medical Biotechnology, College of Life Sciences, Nanjing Normal University, Nanjing, Jiangsu China; 3 Center of Reproductive Medicine, Nanjing Jinling Hospital, Nanjing Medical University, Nanjing, 210002 China; 4 Basic Medical Laboratory, Nanjing Jinling Hospital, Clinical School of Medical College, Nanjing University, Nanjing, 210002 Jiangsu China

**Keywords:** Ageing, Cell division

## Abstract

In recent years, the postponement of childbearing has become a critical social issue. Male fertility is negatively associated with age because of testis aging. Spermatogenesis is impaired with age, but the molecular mechanism remains unknown. The dynamic posttranslational modification O-linked N-acetylglucosamine (O-GlcNAc), which is a type of monosaccharide modification, has been shown to drive the process of aging in various systems, but it has not yet been investigated in the testis and male reproductive aging. Thus, this study aims to investigate the alteration of O-GlcNAc with aging and explore the role of O-GlcNAc in spermatogenesis. Here, we demonstrate that the decline in spermatogenesis in aged mice is associated with elevation of O-GlcNAc. O-GlcNAc is specifically localized in differentiating spermatogonia and spermatocytes, indicating its crucial role in meiotic initiation and progression. Mimicking the age-related elevation of O-GlcNAc in young mice by disabling O-GlcNAcase (OGA) using the chemical inhibitor Thiamet-G can recapitulate the impairment of spermatogenesis in aged mice. Mechanistically, the elevation of O-GlcNAc in the testis leads to meiotic pachytene arrest due to defects in synapsis and recombination. Furthermore, decreasing O-GlcNAc in aged testes using an O-GlcNAc transferase (OGT) inhibitor can partially rescue the age-related impairment of spermatogenesis. Our results highlight that O-GlcNAc, as a novel posttranslational modification, participates in meiotic progression and drives the impairment of spermatogenesis during aging.

## Introduction

Over the past few decades, the average age at which couples reproduce has been delayed significantly, with the mean age of first reproduction now being approximately 30 years old [1]. While increased maternal age is well established to have a negative impact on fertility [2], males can still produce sperm and successfully reproduce even at a very advanced age. However, increasing evidence has suggested that advanced paternal age is related to impaired spermatogenesis, decreased fertility rate and decreased offspring fitness [3]. Although the mechanism underlying age-related reproductive dysfunction in males has drawn great attention in recent years, the precise mechanisms remain unknown.

Spermatogenesis ensures the sustainable production of male gametes, the spermatozoa. The production of haploid cells is achieved through meiosis, a specialized cell division during which a single round of DNA replication is followed by two consecutive chromosome segregations. Meiosis is a complex biological program that is tightly regulated by multiple posttranslational modifications (PTMs), including phosphorylation [4], ubiquitination [5], methylation [6], and SUMOylation [7]. Previous histological studies have reported defects in meiosis in testes from aged men [8]. However, how meiosis is modulated by PTM and how aging affects this modulation are worth exploring.

There are emerging data showing that protein O-GlcNAc, a nutrient-sensitive PTM, regulates diverse cellular processes and drives age-related disease [9]. In contrast to N- and O-glycosylation, O-GlcNAc is a type of monosaccharide modification that cannot be extended to form complex glycan structures and is not restricted to the cell surface and secreted proteins. O-GlcNAc modification exists on nuclear and cytoplasmic proteins; therefore, it is extremely important in nearly every part of cell physiology, including transcription, the cell cycle, and stress responses [10]. O-GlcNAc is a highly dynamic and reversible modification that is regulated by only two enzymes, O-GlcNAc transferase (OGT) and O-GlcNAcase (OGA), which catalyze the addition and removal of O-GlcNAc, respectively. Previous studies in Xenopus oocytes indicated that perturbations of O-GlcNAc impaired female gametogenesis [11], although the precise mechanisms remain elusive. Moreover, how O-GlcNAc is involved in male gametogenesis has not been determined. For this reason, because O-GlcNAc modification plays key roles in many of the major diseases associated with aging [9], we attempted to investigate whether O-GlcNAc represents an important PTM that drives testicular aging.

In this study, we detected elevated O-GlcNAc levels and decreased OGA expression in the aged testis, concomitant with age-related impairment of spermatogenesis. We mimicked the age-related elevation of O-GlcNAc by targeting OGA in young mice and then examined its effect on spermatogenesis. Spermatogenesis is impaired in high-O-GlcNAcylation mice. Furthermore, high O-GlcNAcylation results in increased numbers of DNA double-strand breaks (DSBs) and defects in synapsis and crossing over during the pachytene stage of meiosis I, which further lead to pachytene arrest. Countering the age-related high O-GlcNAcylation partially rescues the impairment of spermatogenesis. Our findings suggest that elevation of O-GlcNAcylation is a driver of testis aging and that maintaining O-GlcNAc homeostasis is essential for appropriate meiotic progression.

## Results

### The localization of O-GlcNAc in the testis suggests its potential function in spermatogenesis

**Fig. 1:**
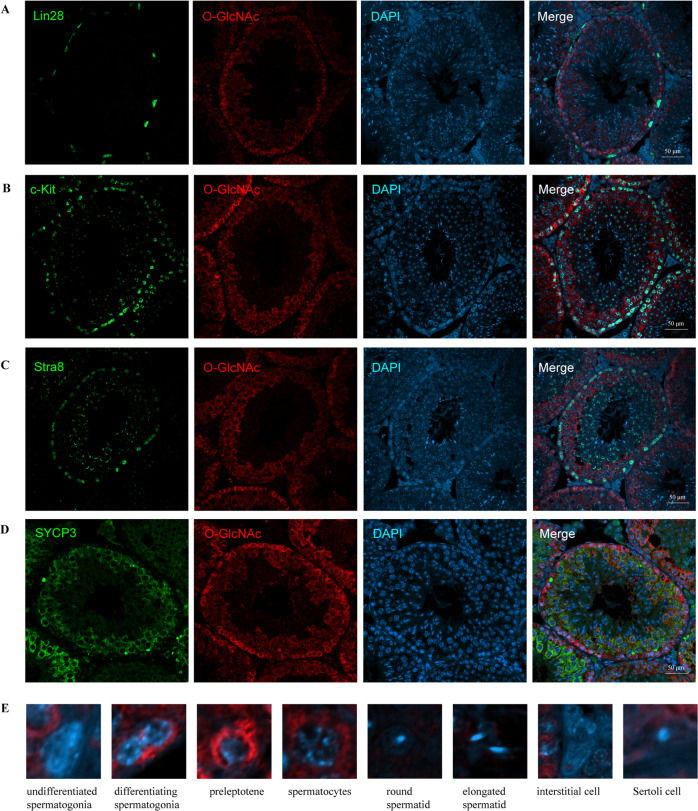
Localization of O-GlcNAc in testes from 2-month-old mice. **A** Immunofluorescence of O-GlcNAc with Lin28 in testicular sections. **B** Immunofluorescence staining of O-GlcNAc with c-Kit. **C** Immunofluorescence staining of O-GlcNAc with Stra8. **D** Immunofluorescence staining of O-GlcNAc with SYCP3. **E** O-GlcNAcylation in different types of testicular cells. Scale bar = 50 μm.

The role of O-GlcNAc in the testis remains unknown. Therefore, to begin our study, we first assessed the localization of O-GlcNAc by co-staining testis sections from 2-month-old mice with O-GlcNAc antibody RL2 and spermatogenic cell-specific antibodies (Fig. 1A–D). Lin28 is a marker of undifferentiated spermatogonia [12]. C-Kit and Stra8 are localized in differentiating spermatogonia, as well as in preleptotene spermatocytes [13, 14]. SYCP3 is specifically localized in spermatocytes [15]. Based on the spermatogenic marker staining and nuclear morphology, we characterized the detailed localization of O-GlcNAc in testicular cells. As shown in Fig. 1E, O-GlcNAc was observed at a high level in differentiating spermatogonia and spermatocytes but was found at low levels or apparently absent in undifferentiated spermatogonia, round spermatids, elongated spermatids, Sertoli cells and interstitial cells. Taken together, these results suggest that O-GlcNAc might specifically participate in the differentiation of spermatogonia, meiotic initiation and progression under physiological conditions.

### Impaired spermatogenesis was accompanied by elevated O-GlcNAc in aged mice

To detect whether testis function is impaired with age, sperm quality was compared between young (2-month-old) and aged (16-month-old) mice. The thickness of the seminiferous epithelium was significantly decreased in the aged group, indicating impairment of spermatogenesis (Fig. S1). The aged mice showed a sharp decrease in testis organ index, sperm concentration and progressive motility (Fig. S1).

Meiosis is a key process required for spermatogenesis. To further assess whether meiosis was altered in aged mice, meiotic chromosome spreads were prepared from testes, followed by immunofluorescence staining for γH2AX and the synaptonemal complex (SC) component SYCP3. Leptotene, zygotene, pachytene and diplotene spermatocytes were distinguished as previously reported [16]. γH2AX, a histone variant that marks DNA DSBs, reveals whether DSBs and their repair are normally initiated and completed. In the testes from young mice, γH2AX accumulated on chromosomes at leptotene and zygotene and was removed from autosomes during the pachytene stage, as expected. However, in the testes of aged mice, γH2AX was improperly persistent on autosomes of pachytene spermatocytes more frequently (Fig. S2). Unrepaired DSBs in pachytene trigger the downstream DNA damage repair system and block cell cycle progression [17]. Therefore, we quantified the cell cycle progression of spermatocytes. In aged mice, the proportion of pachytene spermatocytes was significantly increased, while the proportion of diplotene spermatocytes was decreased, indicating that the spermatocytes were arrested in pachytene (Fig. S2). These data suggest that spermatogenesis is impaired in aged mice, which is associated with meiotic arrest at pachytene/diplotene.

**Fig. 2:**
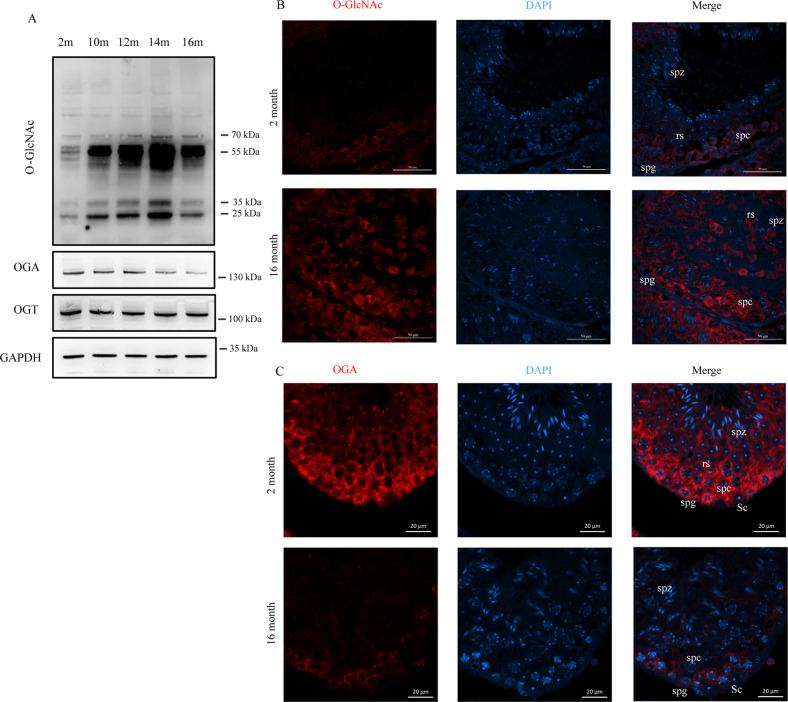
Elevation of O-GlcNAc in the testis during aging. **A** Western blot images of O-GlcNAcylation, OGA and OGT from whole testicular lysates of mice at different ages. GAPDH was used as the internal reference. **B** Representative immunofluorescence images of testicular slices stained with O-GlcNAc. **C** Representative immunofluorescence images of testicular slices stained with OGA. spg spermatogonia, spc spermatocytes, rs round spermatids, spz spermatozoa, Sc Sertoli cells.

Accumulated evidence suggests that O-GlcNAcylation is involved in multiple age-related diseases, including diabetes, cancer and neurodegenerative disease [9]. To identify whether O-GlcNAcylation changes during aging in testes, we first compared the levels of O-GlcNAc in testes from 2-, 10-, 12-, 14- and 16-month-old mice. Western blot images showed that O-GlcNAc levels increased with age and peaked at 14 months. Meanwhile, the protein level of OGA decreased with age, while OGT remained unchanged (Fig. 2A). Immunofluorescence staining analysis demonstrated that in addition to its elevated level, the localization of O-GlcNAc was also altered. In the testes of young mice, O-GlcNAcylation existed in spermatogonia and spermatocytes but disappeared in round spermatids. In the testes of aged mice, O-GlcNAcylation existed not only in spermatogonia and spermatocytes but also in round spermatids (Fig. 2B). Immunofluorescence staining also confirmed the decrease in OGA in aged mice. OGA in the testes of young mice was broadly present in every cell type in the seminiferous tubules, except Sertoli cells. However, the localization of OGA was altered in aged mice, with sharply decreased levels in spermatocytes, round spermatids, and spermatozoa (Fig. 2C). Additionally, OGT located broadly in seminiferous tubules, and its expression level and location did not change in aged mice (Fig. S3A). In summary, these data raise the possibility that the elevation of O-GlcNAc in spermatogenic cells is associated with the impairment of spermatogenesis in aged mice.

### The altered expression pattern of O-GlcNAc and OGA in meiosis during aging is accompanied by meiotic arrest

**Fig. 3:**
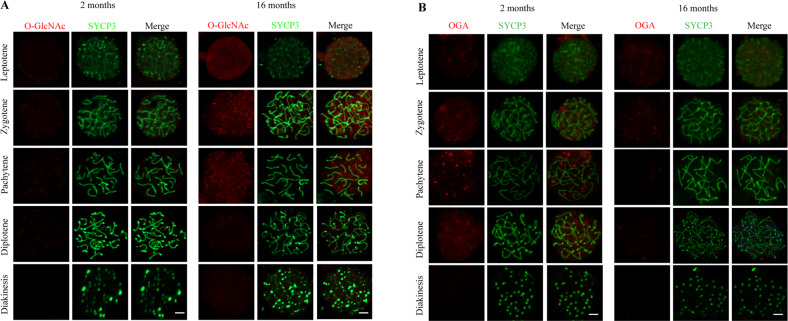
Altered expression patterns of O-GlcNAc and OGA in spermatocytes of young and aged mice. **A** Immunostaining of O-GlcNAc (red) and SYCP3 (green) in prophase spermatocytes prepared from the testes of young (left) and old (right) mice. **B** Immunostaining of OGA (red) and SYCP3 (green) in prophase spermatocytes prepared from the testes of young (left) and old (right) mice. Scare bar = 5 μm.

Given the above finding that spermatocytes are the major O-GlcNAcylated cell population in the testis, we further assessed whether the O-GlcNAc cycling pattern in spermatocytes was altered during aging. Chromosome spreads were prepared to evaluate the changes in O-GlcNAc from leptotene to diakinesis. We did not detect the O-GlcNAcylation signal in prophase spermatocytes from young mice. In the spermatocytes from aged mice, however, O-GlcNAc was elevated; its distribution was presented as diffuse in leptotene and punctate aggregates in zygotene and pachytene, but it disappeared in diplotene and diakinesis (Fig. 3A). Next, we examined the expression of OGA in prophase spermatocytes. In the young mouse group, OGA was elevated and gradually aggregated from leptotene to pachytene, decreased in diplotene and disappeared in diakinesis. However, this pattern was absent in spermatocytes from the aged mouse group (Fig. 3B). The intensity of O-GlcNAcylation and OGA during each stage of prophase in young and old mice is shown in Fig. S3B. Specifically, OGA colocalized with SYCP3, indicating the possible role of O-GlcNAc cycling in synapsis. Based on these data, we speculate that the low level of O-GlcNAc is maintained by OGA in spermatocytes of young mice. The maintenance of this situation is dysfunctional and accompanied by impairment of meiotic arrest in aged mice.

### Mimicking the age-related elevation of O-GlcNAc in young mice impairs spermatogenesis

**Fig. 4:**
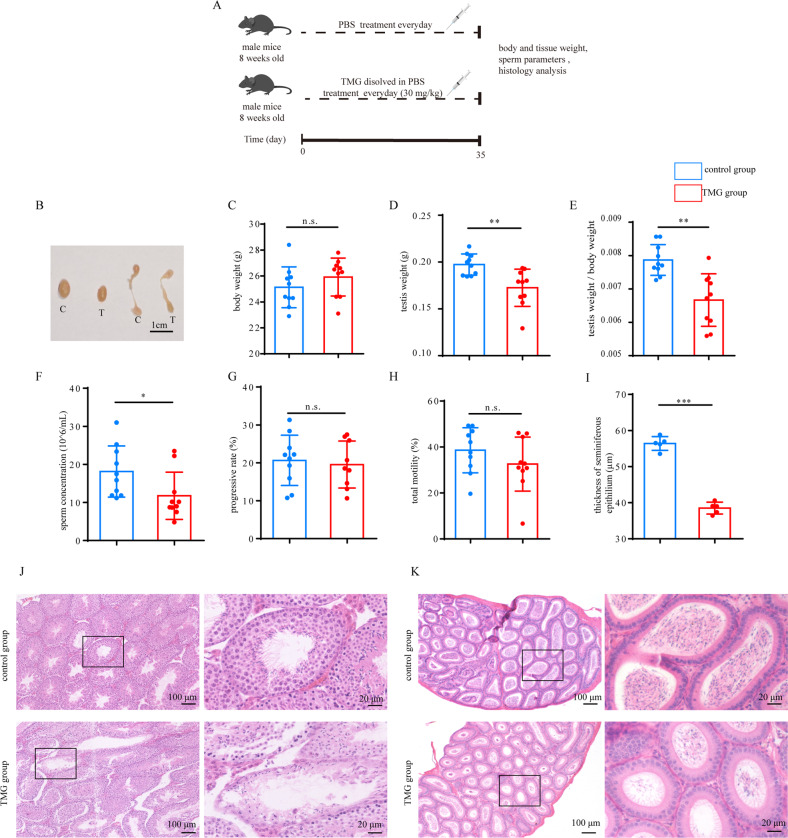
Elevation of O-GlcNAc in young mice elicits aging-associated subfertility. **A** Schematic diagram of elevated O-GlcNAcylation in young mice. **B** Testis and epididymis of control mice and Thiamet-G-treated mice. **C**–**E** Testis weight, body weight and organ index of control mice and Thiamet-G-treated mice. **F**–**H** The concentration, progressive motility and total motility of sperm collected from the caudal epididymis of control and Thiamet-G-treated mice were evaluated using CASA. *N* = 10. **J**, **K** H&E-stained sections of seminiferous tubules and epididymis from control and Thiamet-G-treated mice. **I** Measurements of the thickness of the seminiferous epithelium of control and Thiamet-G-treated mice. Two hundred tubules per testis were examined. Means ± SD. *p < 0.05, **p < 0.01, ***p < 0.001.

We next assessed whether elevation of O-GlcNAc elicited features of impaired spermatogenesis of aging. We constructed a high O-GlcNAcylation mouse model using Thiamet-G (TMG), a widely used OGA inhibitor that potently enhances protein O-GlcNAcylation [18]. According to previously reported effective doses of TMG [19], mice were intraperitoneally injected with 10, 20, 30, or 80 mg/kg TMG and sacrificed after 8 h. Because 30 mg/kg TMG effectively elevated O-GlcNAc (Fig. S4), this dose was selected for further experiments. Mice were treated with TMG for 35 days, which is the duration of the spermatogenic cycle in mice. After 35 days, spermatogenesis-related indicators were detected (Fig. 4A). O-GlcNAcylation increased steadily in the TMG-treated mouse group (referred to as the TMG group) (Fig. S4). Mice in the TMG group did not differ in weight from those in the control group. The testis and epididymis size, testis weight and testis organ index were decreased in the TMG group (Fig. 4B–E). High O-GlcNAcylation also led to a decline in sperm concentration but had no influence on progressive or total sperm motility (Fig. 4F–H). Histologically, high O-GlcNAcylation led to a thinner seminiferous epithelium and fewer sperm in epididymal tubules (Fig. 4I–K). In addition, intraperitoneal administration did not impair the levels of follicle stimulating hormone (FSH), luteinizing hormone (LH), testosterone or inhibin B (Fig. S5), which suggests that the function of the hypothalamus–pituitary–gonadal axis remained normal. Therefore, the impairment of spermatogenesis is caused by in situ factors rather than hormones. These data demonstrate that elevation of O-GlcNAc impairs spermatogenesis in mice.

To further investigate which stages of spermatogenesis were affected by elevated O-GlcNAc, H&E staining was performed and the numbers of round spermatids, spermatocytes and spermatogonia were counted. The proportion of round spermatids decreased, while that of spermatocytes and spermatogonia increased (Fig. S6A). Additionally, the mRNA levels of genes which mark the round spermatids and elongated spermatids decreased (Fig. S6B). These results imply that the transition from spermatocytes to round spermatids might be impaired.

### Elevation of O-GlcNAc leads to defective meiosis with pachytene arrest

**Fig. 5:**
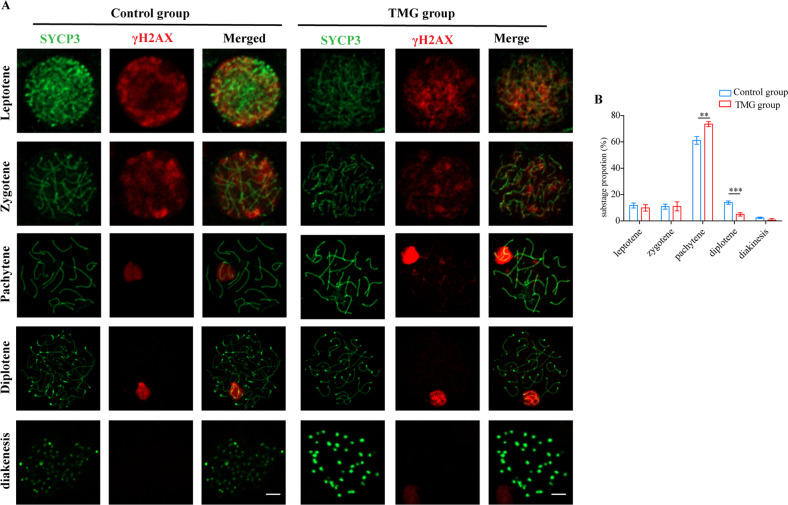
High O-GlcNAcylation impairs the progression of meiosis. **A** Immunostaining of SYCP3 (green) and γH2AX (red) in spermatocytes from control mice (left) and Thiamet-G-treated mice (right). Scare bar = 5 μm. **B** The proportion of cells in each meiotic stage in the testes of control and Thiamet-G-treated mice. Two hundred spermatocytes were evaluated in each testis. The proportion of each substage of spermatocyte is calculated by dividing the number of such cells by the total number of spermatocytes. *N* = 5. Means ± SD. *p < 0.05, **p < 0.01, ***p < 0.001.

Based on the previously described results, we speculated that elevation of O-GlcNAc could lead to defective meiosis. To elucidate the effect of O-GlcNAc on meiosis, chromosome spreads were prepared. In pachytene spermatocytes of control mice, γH2AX staining was restricted to the sex body regions. However, pachytene spermatocytes of TMG group mice exhibited abnormal localization of γH2AX on autosomes, which indicated the failure of DNA damage repair (Fig. 5A). The proportion of pachytene cells was elevated, while that of diplotene cells was decreased, in TMG group mice, indicating arrest at the pachytene stage (Fig. 5B). Consistent with the observed meiotic arrest, more spermatocytes underwent apoptosis, as revealed by TUNEL staining (Fig. S7). These findings suggest that elevation of O-GlcNAc causes failure of DSB repair and leads to pachytene arrest.

### Elevation of O-GlcNAc impairs recombination, crossover formation and synapsis in spermatocytes

**Fig. 6:**
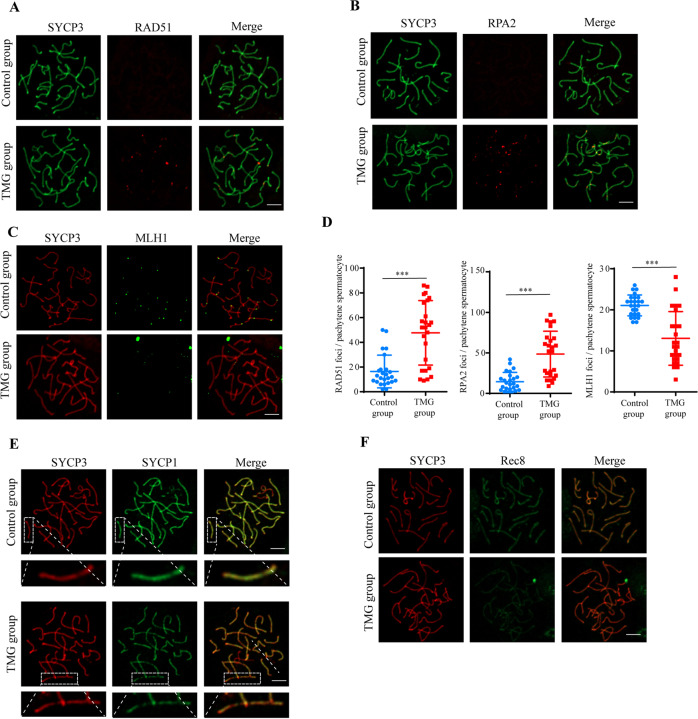
High O-GlcNAcylation impairs recombination and synapsis. **A** Immunostaining of SYCP3 (green) and RAD51 (red) in spermatocytes from control mice and Thiamet-G-treated mice. **B** Immunostaining of SYCP3 (green) and RPA2 (red). **C** Immunostaining of SYCP3 (red) and MLH1 (green). **D** Quantification of RAD51, RPA2, and MLH1 foci in spermatocytes. 30 spermatocytes were evaluated in each group. **E** Immunostaining of SYCP3 (red) and SYCP1 (green). **F** Immunostaining of SYCP3 (red) and Rec8 (green). Means ± SD. *p < 0.05, **p < 0.01, ***p < 0.001.

Defective recombination is a significant mechanism of pachytene arrest [20]. Successful recombination relies on the proper repair of DSBs. Therefore, we investigated several markers that are involved in DNA repair and recombination, including RPA2, RAD51 and MLH1. RPA2 and RAD51 are reported to bind to the DNA break site [21, 22]. Spermatocytes in control mice lost RPA2 and RAD51 foci in pachytene, indicating the repair of DSBs in this stage, whereas in TMG mice, these foci were retained on the autosomes of pachytene spermatocytes (Fig. 6A, B, D). MLH1 marks the formation of crossovers during meiotic recombination. While at least one crossover per homologous chromosome pair was observed in pachytene spermatocytes of control mice, the number of MLH1 foci in high O-GlcNAc mice was significantly reduced, suggesting that meiotic recombination was impaired (Fig. 6C, D). Homologous recombination is strictly regulated by and highly dependent on the proper formation of the SC [23]. Therefore, we further assessed synapsis in control and high-O-GlcNAc mice. SYCP1, which forms the transverse filament of the SC, produced a discontinuous signal along the chromosome axis in spermatocytes of high O-GlcNAc mice (Fig. 6E). Consistently, the signal of REC8, which is a component of the cohesin complex and ensures the formation of the SC, was evidently decreased in high O-GlcNAc mice (Fig. 6F). These results suggested that high O-GlcNAcylation might influence synapsis, recombination and crossover formation and further result in pachytene arrest.

### Decreasing O-GlcNAc using OSMI-1 partially rescues spermatogenesis in aged mice

**Fig. 7:**
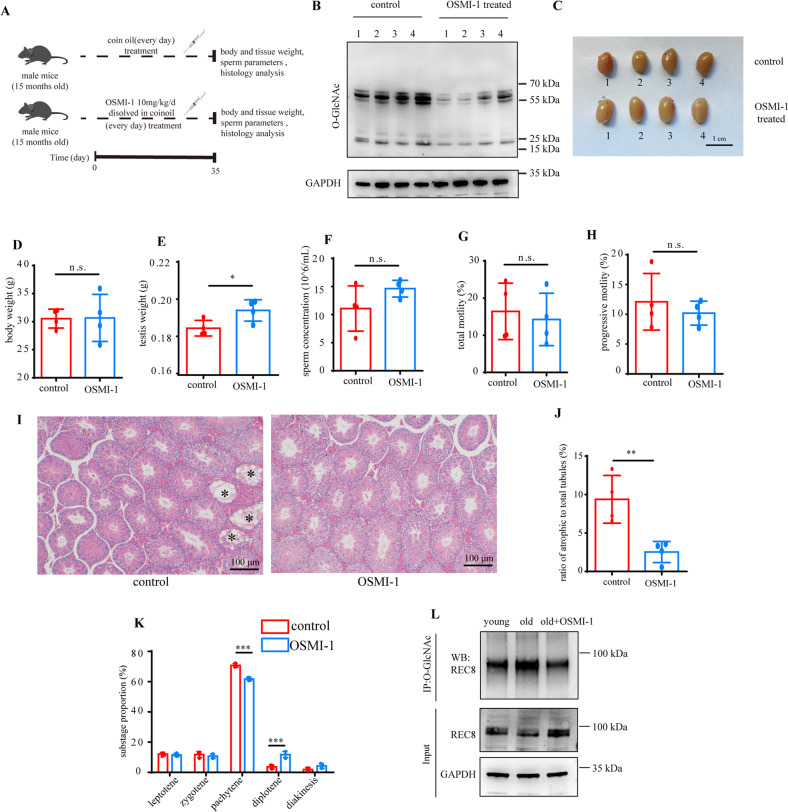
Decreased O-GlcNAc restores spermatogenesis in aged mice. **A** Schematic diagram of decreasing O-GlcNAc using OSMI-1 in aged mice. **B** Western blot images of O-GlcNAc from mouse whole testicular lysates. GAPDH was used as the internal reference. **C** Images of testes from aged control and OSMI-1-treated mice. **D**, **E** Testis weight and body weight of aged control and OSMI-1-treated mice. **F**–**H** Sperm concentration, total motility and progressive motility of sperm collected from the caudal epididymis of aged control and OSMI-1-treated mice, assayed using CASA. *N* = 4. **I** H&E-stained sections of seminiferous tubules from aged control and OSMI-1-treated mice. **J** Statistical analysis of the atrophic tubule ratio in aged control and OSMI-1-treated mice. Two hundred tubules were analyzed in each mouse. Bar = 100 μm. **K** The proportion of cells in each meiotic stage in the testes of control and OSMI-1-treated mice. 200 spermatocytes were evaluated in each testis. The proportion of each substage of spermatocyte is calculated by dividing the number of such cells by the total number of spermatocytes. **L** Proteins from testes of young, old and OSMI-1 treated old mice were immunoprecipitated with O-GlcNAc antibody. O-GlcNAcylated REC8 was detected using REC8 antibody. GAPDH was used as the internal control. Means ± SD. *p < 0.05, **p < 0.01, ***p < 0.001.

To test whether decreasing O-GlcNAc in aged mice can restore spermatogenesis, we used the enzymatic OGT inhibitor OSMI-1, which was previously reported to be suitable for long-term use without side effects in a mouse model [24]. After intraperitoneal administration of 10 mg/kg OSMI-1 in aged mice for 35 consecutive days (Fig. 7A), sperm parameters and testicular histology were evaluated. OSMI-1 effectively decreased O-GlcNAc in aged mice (Fig. 7B). While the body weight remained unchanged, the testis size and testis weight showed an increase in OSMI-1-treated mice (Fig. 7C–E). However, we observed no significant increase in sperm parameters, only a small upward trend in sperm concentrations (Fig. 7F–H). Additionally, an analysis of testicular histology further supported that OSMI-1 restored spermatogenesis in aged mice. The ratio of atrophic tubules to total tubules in OSMI-1-treated mice was decreased compared to that in control aged mice (Fig. 7I, J). The arrested meiosis was rescued in aged mice after treated with OSMI-1 (Fig. 7K). OSMI-1 treatment down-regulated *Rad51* and *Dmc1*, which suggested that OSMI-1 rescued DSBs in aged testis. Furthermore, the markers of round spermatid, *Acrv1* and *Tex12* elevated in OSMI-1 group (Fig. S8). These results suggest that decreasing O-GlcNAc could partially alleviate the impairment of spermatogenesis in aged mice, thus providing a potential therapeutic target for improving sperm production in aged males. Furthermore, to detect crucial O-GlcNAcylated proteins in the testes of young mice, old mice and OSMI-1 treated old mice, proteins were immunoprecipitated with O-GlcNAc antibody. The O-GlcNAcylated level of REC8 was elevated in old mice compared with young mice, while decreased in OSMI-1 treated old mice. Interestingly, the protein level of REC8 decreased in testes of aged mice but increased in OSMI-1 treated group (Fig. 7L). This result suggest that O-GlcNAcylated REC8 might participate the impairment of spermatogenesis, while the specific mechanism still need to be verified further.

## Discussion

In summary, our research indicates that age-related elevation of O-GlcNAcylation in the testis disturbs key processes in meiosis and thereby contributes to the impairment of spermatogenesis. Mimicking the age-related elevation of O-GlcNAcylation by targeting OGA impairs synapsis and DSB repair and further leads to pachytene arrest in young mice. Additionally, disrupting the O-GlcNAc cycle can partially rescue the impairment of spermatogenesis in aged mice, which provides a potential target for the improvement of sperm production. Ultimately, our research highlights O-GlcNAcylation as a new metabolic-coupled PTM mechanism in the regulation of meiosis and a key mediator of age-related subfertility.

Male reproductive aging and infertility have been deeply discussed over the past several decades. A retrospective study of 4822 semen analyses reported that sperm concentration declines after 40 years of age [25]. The deterioration of sperm production during aging can be attributed to the impairment of testicular histological architecture, presented as thinning of the seminiferous epithelium, reduced vascularization of the testes and narrowing of the tubules [26]. In a morphometric study, young and old men had similar numbers of A and B spermatogonia per gram of testicular parenchyma, while the transition of early primary to late primary spermatocytes was not as efficient in older men [27]. A more recent study reported that the paring, synapsis, and recombination of prophase spermatocytes are affected by age in mice. In the current study, we demonstrate that spermatogenesis is impaired in aged testes and that meiosis is partially arrested at the pachytene stage. Furthermore, DSBs are not fully repaired in pachytene. These results were consistent with previous studies that DSBs elevated in aged testis and led to cell cycle arrest [28, 29]. Cumulatively, these studies and our results suggest that the elevation of DSBs may be one mechanism that causes meiotic arrest and germ cell loss in aged mice.

A previous study reported that the levels of O-GlcNAcylation in various tissues increased during aging, including the brain, lung, skin and thymus [30]. In our study, remarkable O-GlcNAcylation increases and OGA decreases were observed in the testes during aging. Thus, the elevation of O-GlcNAc might be a multisystem phenomenon. Mimicking high O-GlcNAcylation in mice impairs spermatogenesis. Analogous to our findings, the involvement of O-GlcNAc and OGA in regulating oocyte development was also reported in previous studies [31]. Under hyperglycemic conditions, the developmental competence of mouse oocytes was impaired by the O-GlcNAcylation of HSP90 [31]. Moreover, elevation of O-GlcNAcylation by OGA disruption leads to mitotic defects, including cytokinesis failure, lagging chromosomes and binucleation, in embryos [30]. Our results are consistent with these studies, providing further evidence that maintaining O-GlcNAc homeostasis is important for the cell cycle, in both mitosis and meiosis.

O-GlcNAc exhibits specialized localization in differentiating spermatogonia and spermatocytes, which are the cells that are undergoing meiotic initiation and progression. Therefore, we focused particularly on its role in meiosis. During the leptotene phase of meiosis, DSBs are induced by SPO11, a meiosis-specific endonuclease [32]; DSBs are then repaired during zygotene through homologous recombination, which promotes synapsis and paring. At pachytene, DSBs are fully repaired, and synapsis is fully completed. In our study, elevation of O-GlcNAc led to synapsis and recombination defects, which induced pachytene arrest and germ cell loss. The colocalization of O-GlcNAc and OGA with SYCP3 indicates the possible role of O-GlcNAc in synapsis. These data further confirm that meiosis is regulated by O-GlcNAc. Many proteins participating in meiosis, such as SYCP2/3, REC8 and SMC3 are reported to be phosphorylated, and their phosphorylation ensures the normal process of meiosis [33]. Considering the competitive relationship between O-GlcNAcylation and phosphorylation, the elevation of O-GlcNAc in aged testis might impair the phosphorylation which is required for meiosis. Nevertheless, although diverse proteins participating in multiple biological processes are reported to be regulated by O-GlcNAcylation, there has been no detailed investigation of O-GlcNAcylated proteins involved in meiosis. We reviewed the available O-GlcNAcylation data for crucial meiosis-related proteins [34] and presented this information in Table S1. However, most of these proteins were identified in mass spectrometry studies without experimental validation. MDC1, EWSR1, KAT5, SIRT1 and TDP-43 were validated to be O-GlcNAcylated by experiments, but their precise functions in meiosis remain elusive. In this study, we identified one O-GlcNAcylated meiotic protein, REC8, which O-GlcNAcylated level was elevated and protein level was decreased in old mice compared with young mice. Whether O-GlcNAcylation of REC8 influences the protein level and how O-GlcNAcylated REC8 participate in meiosis still need further exploration. Further investigation into protein O-GlcNAcylation in meiosis will broaden our understanding of the PTM landscape in this process.

The elevation of O-GlcNAcylation in young mice produces spermatogenetic defects mimicking those in aged mice, which prompts us to consider O-GlcNAc as a therapeutic target for restoration of sperm production. It is the most appropriate choice to restore OGA in aged mice, as OGA decreases with age in the testis. However, an OGA-selective activator has not yet been developed [35]. As an alternative, we used OSMI-1, which is an effective OGT inhibitor, to balance O-GlcNAc homeostasis in aged testes. Although OSMI-1 didn’t rescue the sperm parameters in aged mice, it increased the testis size and weight, also promoted meiotic progression. The restorative effects of OSMI-1 in spermatogenesis provide a new potential therapy for reproductive aging. In addition, the therapeutic effects of other OGT inhibitors and HBP inhibitors need further exploration.

**Fig. 8:**
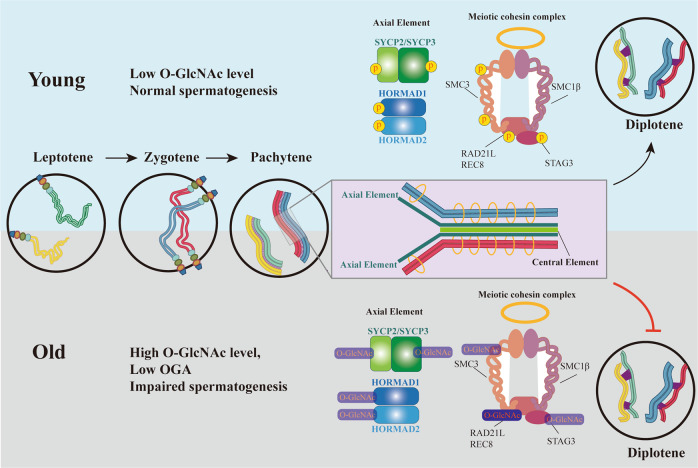
Models summarize the putative mechanisms by which O-GlcNAc is involved in male meiosis in young and old conditions. During leptotene stage, recombination is initiated between homologs chromosomes and synaptonemal complexes (SC) become organized. The homologous chromosomes come into close proximity and SCs start to assemble at zygotene. In the pachytene stage, the homologous chromosomes are fully synapsed and crossovers are formed. SCs disassemble at diplotene and homologous chromosomes are attached to each other at crossover sites. The enlarged structure of SC complex is shown in the pink box. The core protein components of the meiotic chromosome axis, including SYCP2/3, HORMAD 1/2, SMC3, REC8 and STAG3, are reported to be phosphorylated. At the young age, the phosphorylation events promote meiotic progression. Age-related elevation of O-GlcNAc competitive inhibits the phosphorylation signal, leads to the impairment of synapsis and recombination, and further causes the pachytene arrest. The O-GlcNAcylated proteins during meiosis remain to be determined.

A model illustrating the possible mechanism and roles of O-GlcNAc in spermatogenesis of young and aged mice is presented in Fig. 8. In conclusion, our study provides new insight into how O-GlcNAcylation participates in age-related spermatogenic disorders and suggests a new target for improving male sperm production during aging.

## Materials and methods

### Animals and treatments

C57/BL/6 mice were purchased from the Animal Core Facility of Nanjing Medical University. Animals were housed on a 12 h light:12 h dark cycle at 22 ± 2 °C with free access to food and water. Animal experiments were performed with permission from the ethics committee of Nanjing Jinling Hospital. To establish a high-O-GlcNAcylation mouse model in 2-month-old mice, Thiamet-G (APExBIO, Suzhou, China) (dissolved in saline, 30 mg/kg/day) was injected intraperitoneally for 35 days. To reduce O-GlcNAcylation in old mice, OSMI-1 (MedChemExpress, Shanghai, China) was dissolved in corn oil containing 10% DMSO and injected intraperitoneally for 35 days. In the control groups, the same volume of solvent was administered intraperitoneally. No specific exclusion or inclusion used for animal experiments. Randomization and blinding were used in animal experiments.

### Testicular histopathological and immunofluorescence analysis

Mouse testes were fixed in Bouin’s fixative for 12 h and then in 4% paraformaldehyde for 12 h, followed by embedding in paraffin. Paraffin sections were cut at 5 μm. Slides were deparaffinized in xylene, rehydrated by a graded alcohol series, and stained in hematoxylin and eosin solution. For immunofluorescence staining, the slides were incubated in citric acid buffer and heated for 15 min in a microwave for antigen retrieval. After blocking in 3% BSA buffer, sections were incubated with the primary antibody at 4 °C overnight. Then, after washing in TBST 3 times, slides were incubated with secondary antibody for 1 h at room temperature. Finally, the slides were stained with 4′,6-diamidino-2-phenylindole (DAPI). The antibodies used and their dilutions are listed in Table S2. To detect apoptotic cells, terminal deoxyribonucleotide transferase-mediated nick end labeling (TUNEL) was conducted. The slides were washed once with PBS, and then, apoptotic cells were detected by TUNEL via an in situ Cell Death Detection kit (Roche, Basel, Switzerland) according to the manufacturer protocol. Images were captured using an Olympus microscope (IX73; Olympus Corporation, Tokyo, Japan).

### Quantitative real-time PCR

A Total RNA Isolation Kit (BEI-BEI Biotech, Zhengzhou, China) was used to isolate RNA from the testis tissue. To detect mRNA, HiScript III RT SuperMix (Vazyme, Nanjing, China) reverse transcriptase was used to synthetize cDNA from mRNA. Quantitative real-time PCR was performed using SYBR qPCR Master Mix (Vazyme, Nanjing, China) with gene-specific forward and reverse primers performed by Roche LightCycler® 96 Real-time PCR system (Roche Diagnostics, Basel, Switzerland). Real-time PCR was performed in triplicate. GAPDH was used as an endogenous control. The relative expression level was calculated using the comparative ΔΔCt method. The sequences of the primers are listed in Table S3.

### Computer‑assisted sperm analysis (CASA)

CASA was conducted as described previously [36]. Briefly, the epididymis was dissected in human tubal fluid medium, and the sperm inside were squeezed out with forceps. After removing the tissue fragments, the sperm were incubated at 37 °C for 30 min. The sperm were then subjected to motility analyses using a Sperm Quality Analyzer. For each measurement, the spermatozoa suspension was loaded into a microchamber slide with 100 μm depth. Three hundred spermatozoa were analyzed using the standard setting.

### Examination of serum hormones

Blood samples were collected from mice, and serum was obtained by centrifugation for 10 min at 1000 × *g.* Serum testosterone, FSH and LH were measured using commercial ELISA kits (Shanghai MLBIO Biotechnology Co. Ltd, Shanghai, China) according to the manufacturer’s instructions. Briefly, microtiter plate wells were coated with purified horseradish peroxidase (HRP)-labeled mouse sex hormone antibodies. After the samples were combined with the antibodies, 3,3′,5,5′-tetramethylbenzidine (TMB) substrate solution was added and reacted with HRP. Sulfuric acid solution was added to terminate the reaction, and the color change was measured spectrophotometrically at a wavelength of 450 nm. The concentrations of sex hormones in the samples were then determined by comparing the O.D. values of the samples to a standard curve.

### Preparation of meiotic chromosome spreads

Meiotic chromosome spreads were prepared as previously described [37]. Briefly, after isolating testis from mice, the tunica albuginea were removed from testis, and tubules were submerged in a hypotonic extraction buffer (30 mM Tris-HCl, 50 mM sucrose, 17 mM trisodium citrate dihydrate, 5 mM ethylenediaminetetraacetic acid, 0.5 mM dithiothreitol, and 0.5 mM phenylmethylsulfonyl fluoride, pH 8.2) for 45–60 min. The tubules were dissected in 100 mM sucrose to release cells and then pipetted to suspend the released cells. The cell suspension was then spread on a slide containing 1% PFA and 0.15% Triton X-100. The slides were incubated in a covered humidified chamber at room temperature for 2.5 h and then air dried. Finally, the slides were washed with PBST and stained with antibodies for analysis. The antibodies used and their dilutions are listed in Table S2.

### Western blotting

Protein was extracted from testicular tissue samples using RIPA (radioimmunoprecipitation assay) lysis buffer containing protease inhibitor cocktail (Roche, Mannheim, Germany) and 1 mM phenylmethylsulfonyl fluoride (PMSF) (Beyotime, Shanghai, China). After measuring the protein concentrations, 20 μg protein was loaded onto 4–20% gels (ACE Biotechnology, Jiangsu, China) in each well and then transferred to a polyvinylidene fluoride (PVDF) membrane. After transfer, 5% BSA was used to block the membranes for 1 h at room temperature. The membranes were incubated with primary antibodies diluted according to the manufacturer’s protocols in Western antibody diluent buffer (NCMbiotech, Suzhou, China) incubate overnight at 4 °C. After three washes with 0.1% Tween-20 in TBS (TBST), the membranes were incubated with HRP-labeled secondary goat anti-rabbit or goat anti-mouse antibody for 1 h at room temperature. Images were captured using an ECL Plus Western Blotting Detection System (Tanon 5200). The antibodies used and their dilution ratios are listed in Table S2.

### Statistical analysis

All data were analyzed in SPSS 19.0 Software. The biological repeats are indicated by ‘n’ in the figure legends. The results were recorded as the means ± SDs. Sample size was not estimated. Bartlett’s test was used to determine whether the variances between several groups are equal. If the variances are similar between experimental groups, two-tailed unpaired Student’s *t* test was used. *P* < 0.05 was considered to indicate statistical significance.

## Supplementary information

Change of authorship

Supplementary figure 1

Supplementary figure 2

Supplementary figure 3

Supplementary figure 4

Supplementary figure 5

Supplementary figure 6

Supplementary figure 7

Supplementary figure 8

Supplementary table

Supplementary Figure Legend

Original Data File

## Data Availability

The data that support the findings of this study are available from the corresponding author upon reasonable request.
