# Supplementary figures and images for: Age-related elevation of O-GlcNAc causes meiotic arrest in male mice
Source: Cell Death Discov. 2023 May 15;9:163. doi: 10.1038/s41420-023-01433-x (PMC10185674; doi:10.1038/s41420-023-01433-x)

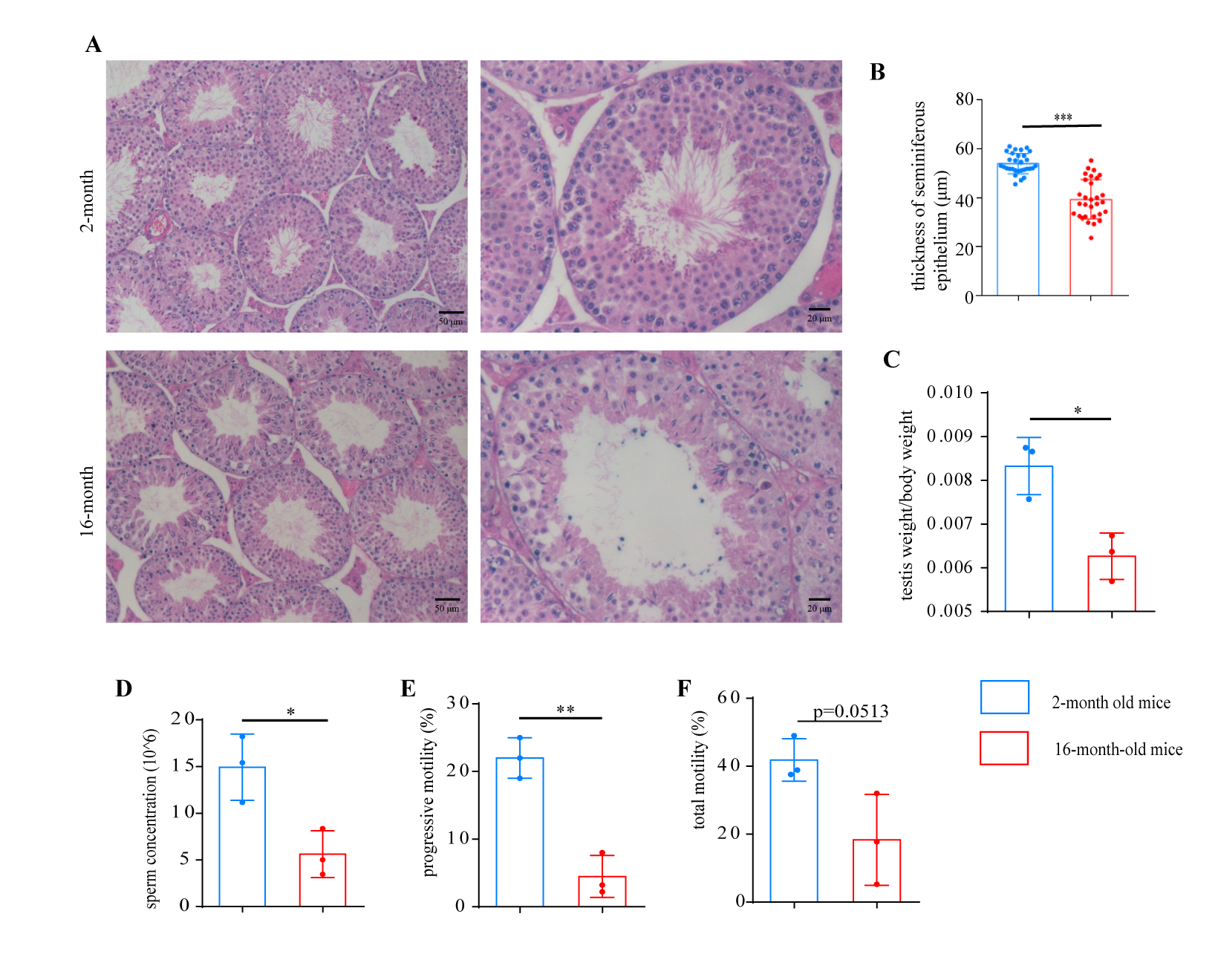

Supplement: Supplementary file 2 — Supplementary figure 1 [file 41420_2023_1433_MOESM2_ESM.tif]

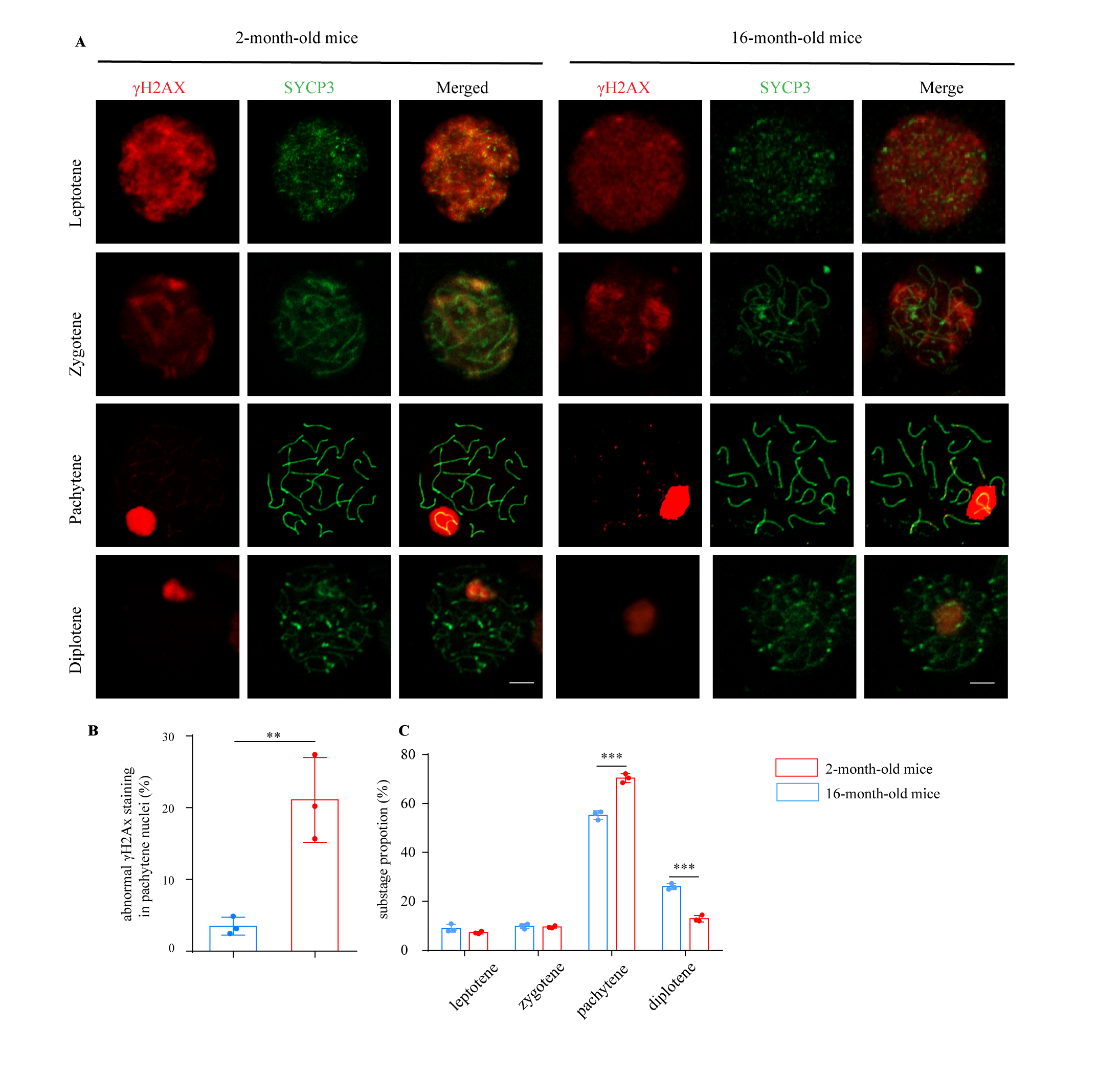

Supplement: Supplementary file 3 — Supplementary figure 2 [file 41420_2023_1433_MOESM3_ESM.tif]

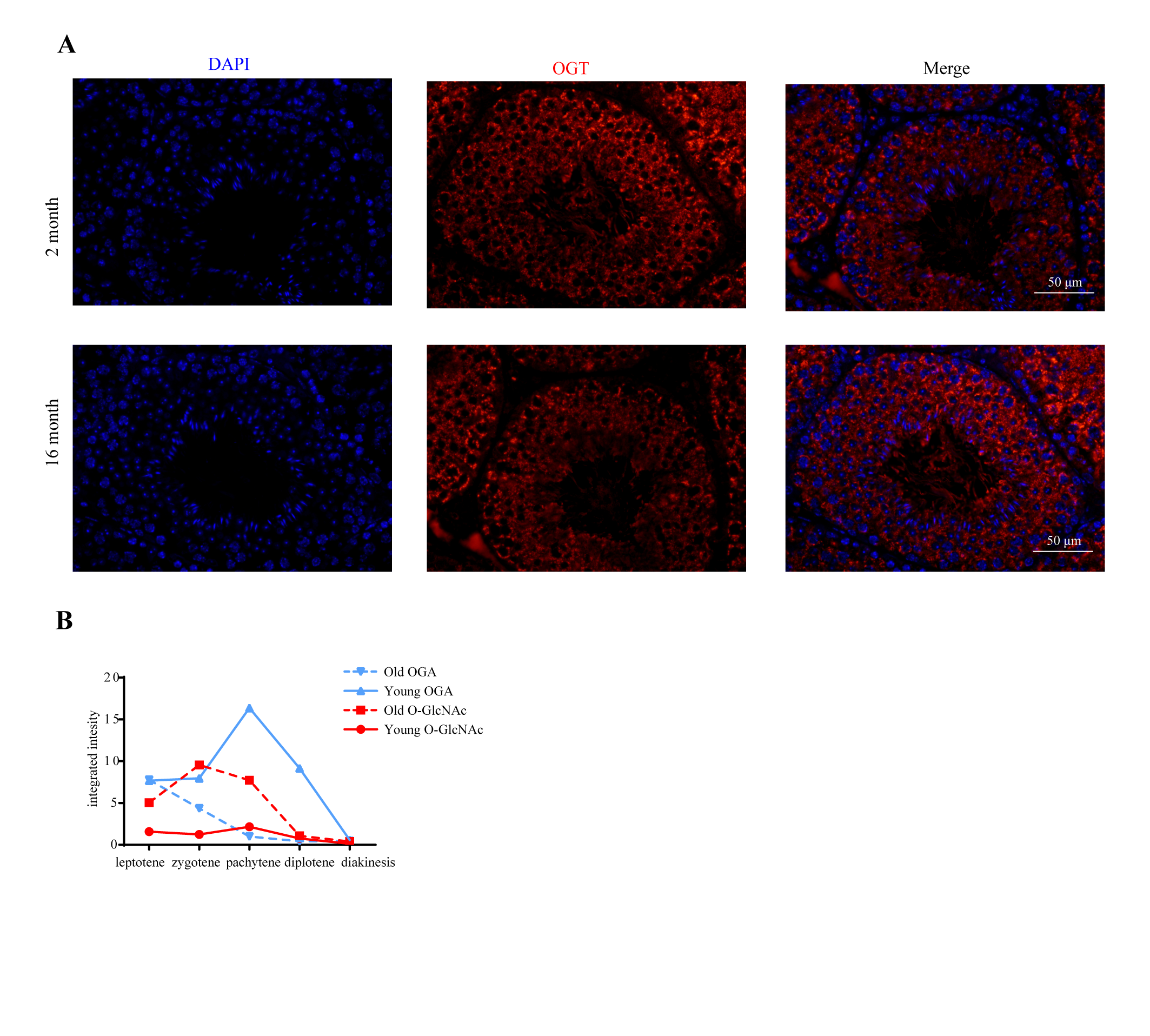

Supplement: Supplementary file 4 — Supplementary figure 3 [file 41420_2023_1433_MOESM4_ESM.tif]

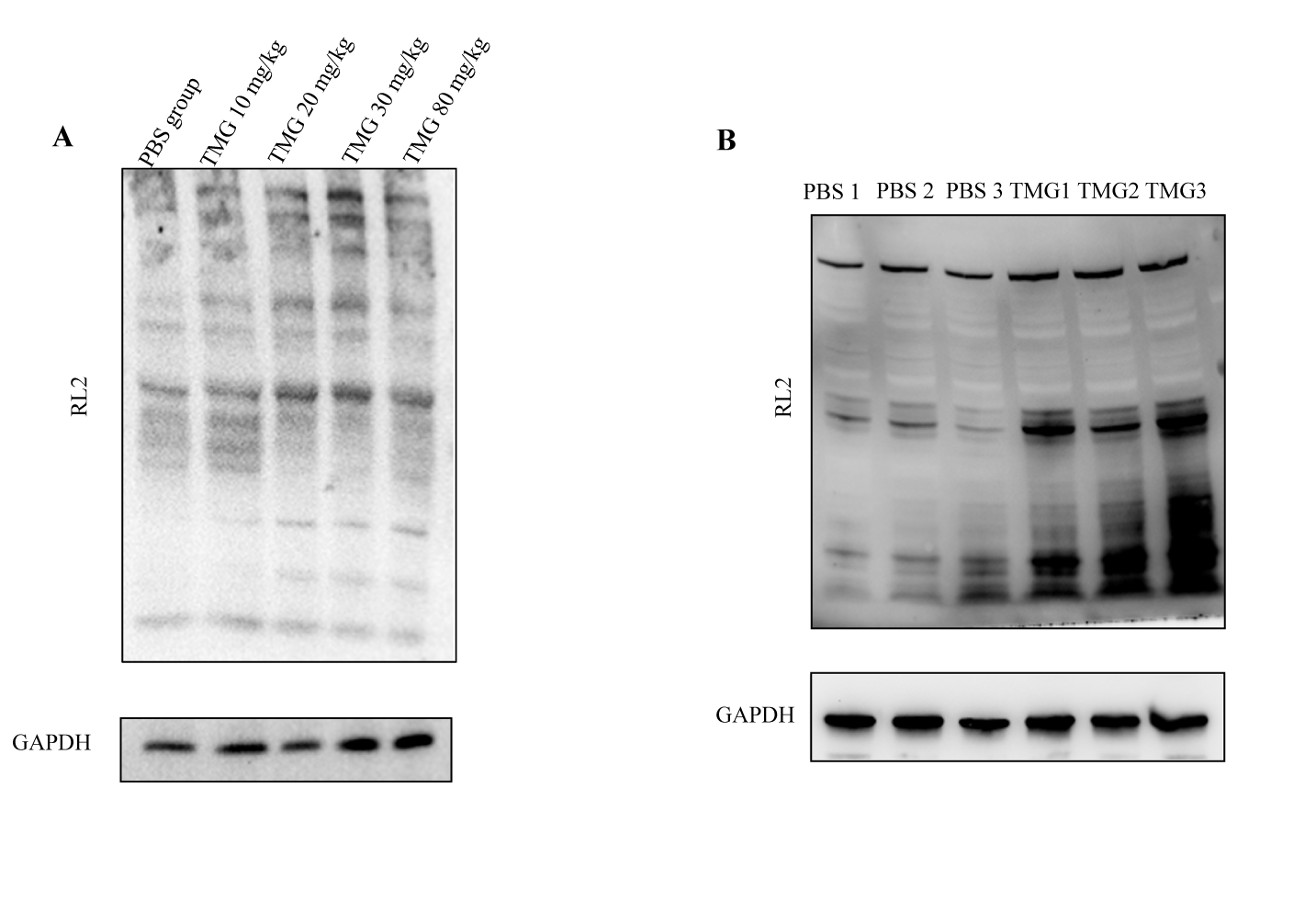

Supplement: Supplementary file 5 — Supplementary figure 4 [file 41420_2023_1433_MOESM5_ESM.tif]

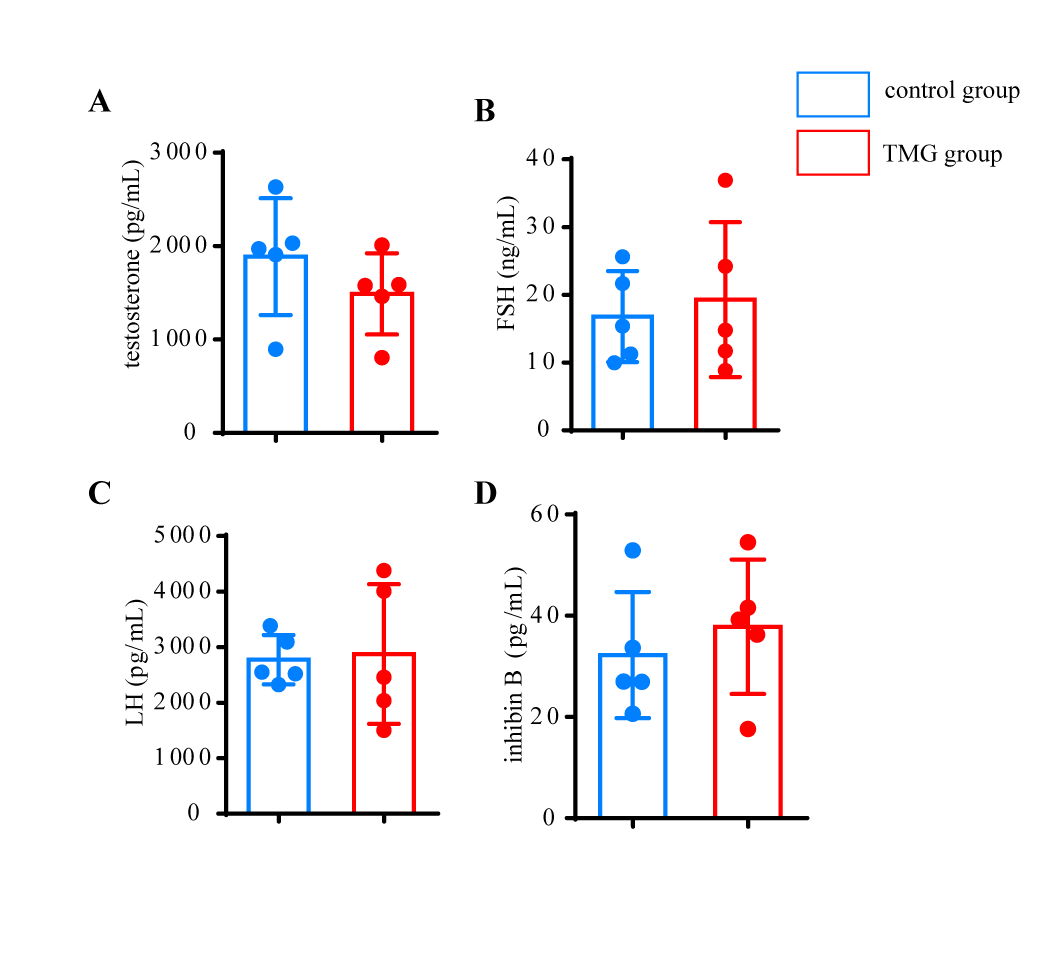

Supplement: Supplementary file 6 — Supplementary figure 5 [file 41420_2023_1433_MOESM6_ESM.tif]

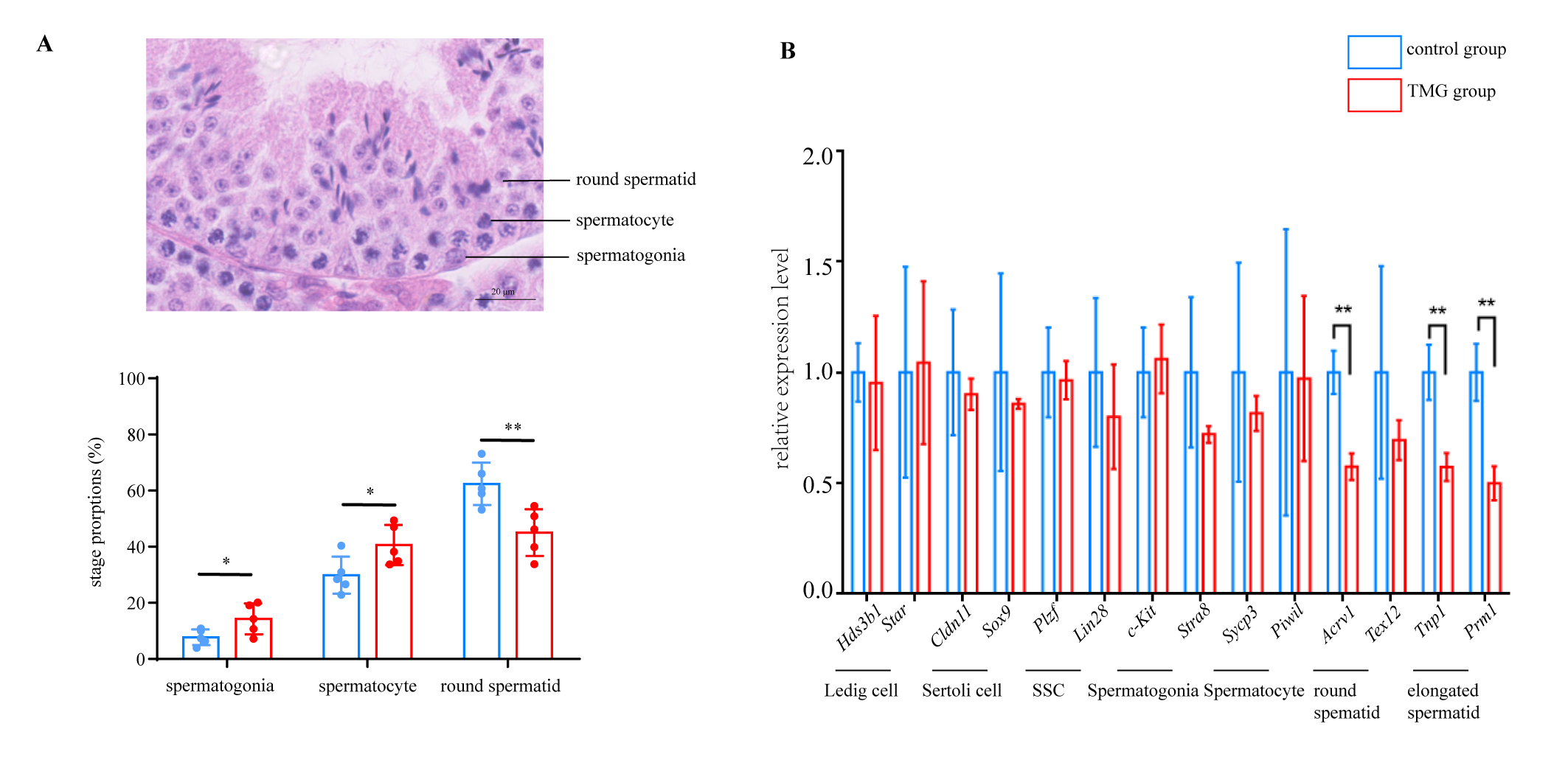

Supplement: Supplementary file 7 — Supplementary figure 6 [file 41420_2023_1433_MOESM7_ESM.tif]

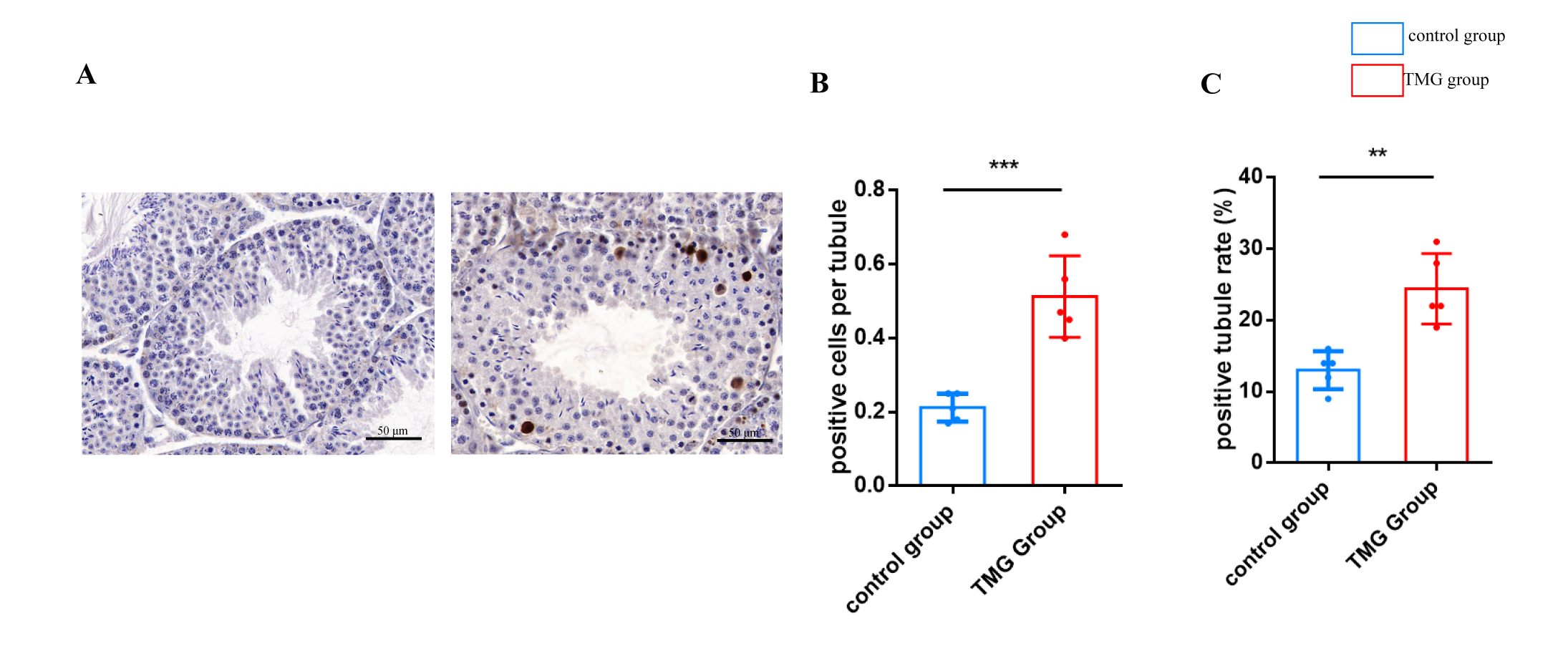

Supplement: Supplementary file 8 — Supplementary figure 7 [file 41420_2023_1433_MOESM8_ESM.tif]

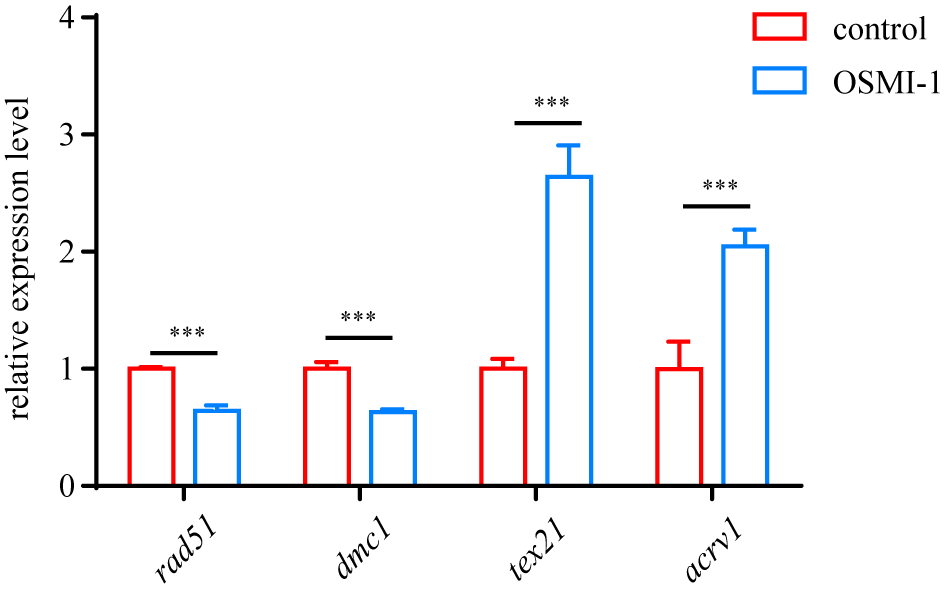

Supplement: Supplementary file 9 — Supplementary figure 8 [file 41420_2023_1433_MOESM9_ESM.tif]
