# Supplementary material for: Age-related elevation of O-GlcNAc causes meiotic arrest in male mice
Source: Cell Death Discov. 2023 May 15;9:163. doi: 10.1038/s41420-023-01433-x (PMC10185674; doi:10.1038/s41420-023-01433-x)
Supplement: Supplementary file 10 — Supplementary table [file 41420_2023_1433_MOESM10_ESM.docx]

***T******able. S1 O-GlcNAcylated proteins in meiosis***

| Protein name | Meiotic role | O-GlcNAc site | validation | Reference |
| --- | --- | --- | --- | --- |
| ATR | DNA damage sensing | S2075,S2414 | Mass spectrometry | (39) |
| ATRIP | Interacts with ATR |  | Mass spectrometry | (40) |
| FBX47 | Regulates the stability of the telomeric shelterin complex | S65 | Mass spectrometry | (39) |
| KASH5 | Homolog pairing and recombination | T189 | Mass spectrometry | (41) |
| MDC1 | Required for expanding gamma-H2Ax along the entire unsynapsed chromatin region | S72, S108, S112, T1116, S1132, T1133, T1138, S1337, S1417, S1419, T1582, S1583 | Mass spectrometry and experimental validation | (42) |
| PDS5A | Regulates the lenth of meiotic chromosome axis and telomere integrity | T170, S865, S1097 | Mass spectrometry | (43) |
| PDS5B | Regulates the lenth of meiotic chromosome axis and telomere integrity | S140, T518 | Mass spectrometry | (44) |
| RAD18 | Required for maintaining MSCI | S130, S164, T468 | Mass spectrometry | (45) |
| RAD21 | Required for establishing cohesion in the premeiotic S phase |  | Mass spectrometry | (46) |
| REC8 | Required for sister chromatid cohesin |  | Mass spectrometry | (39) |
| SCML2 | Required to estabilish the unique epigenome of meiotic sex chromosomes | T567, S568, S570, S574 | Mass spectrometry | (47,48) |
| SETDB1 | Facilitates the sex chromosome remodeling. Required for establishing MSCI |  | Mass spectrometry | (45,46) |
| SGOL2 | Regulates meiotic centromeric cohesin |  | Mass spectrometry | (39) |
| SIX6OS1 | Required to initiate the assembly of the synaptonemal complex |  | Mass spectrometry | (39) |
| SKP1 | Required for TRIP13 abundance or stability |  | Mass spectrometry | (45,49) |
| SMC1α | Required for establishing sister chromatid cohesion during the premeiotic S-phase | T722, S882 | Mass spectrometry | (50,51) |
| SMC1β | Required for normal chromosome axis lenth and telomere integrity | T815 | Mass spectrometry | (39) |
| Sororin | Regulates the cohesion complex |  | Mass spectrometry | (40) |
| STAG3 | Forms complex with SMCα/β. Involved in sister formation of chromatid cohesion. |  | Mass spectrometry | (39,40) |
| SUN1 | Required for telomere dynamic movement and homologous chromosome pairing and recombination | T449, T590, T590 | Mass spectrometry | (47) |
| SYCE1 | Required to initiate the assembly of the synaptonemal complex |  | Mass spectrometry | (40,52) |
| SYCP1 | Provides the structural basis for meitic chromosome synapsis |  | Mass spectrometry | (39) |
| SYCP2 | Required for synaptonemal complex assembly and synapsis |  | Mass spectrometry | (39) |
| TOPBP1 | Involved in activating ATR and establishing MSCI |  | Mass spectrometry | (53) |
| TRIP13 | Required to remove HORMAD1/2 from the synapsed chromosome axis |  | Mass spectrometry | (49) |
| ANKRD31 | Prevents recombination initiation at promoters and ensures recombination in the PAR |  | Mass spectrometry | (39) |
| ATM | Required for first wave of H2AX phosphorylation. DNA damage sensing. | S601, S941, S1731, T2608 | Mass spectrometry | (54) |
| CDYL | Interacts with PDM9 |  | Mass spectrometry | (46) |
| CXXC1 | Participates in linking H3K4me3 sites to DSB machinery on the chromosome axis |  | Mass spectrometry | (55) |
| EHMT2 | Forms complex with PRDM9 and CDYL in preleptotene and leptotene |  | Mass spectrometry | (46) |
| EWSR1 | Required to tether DSB hotspots to DSB machinery on the chromosome axis |  | Mass spectrometry and experimental validation | (56,57) |
| HELLS | Required for correct placement and repair of DSBs |  | Mass spectrometry | (40) |
| IHO1 | Required for DSB formation and regulation of DSB number | S18, S34 | Mass spectrometry | (44) |
| KAT5 | Required for regulating chromosome axis length and DSB numbers | S119 | experimental validation | (56) |
| MEI1 | Required for DSB formation | S760 | Mass spectrometry | (39) |
| PRDM9 | Required for positioning of DSB hotspots |  | Mass spectrometry | (39,40) |
| SIRT1 | Required for inhibiting meiotic DSB in rDNA in yeast | S549, T614 | Mass spectrometry and experimental validation | (45,58–60) |
| BLM | Required for DSB end resectioning and noncrossover formation |  | Mass spectrometry | (39) |
| BRCA2 | Required for loading RAD51 and DMC1 onto RPA-binding ssDNA |  | Mass spectrometry | (39,54) |
| CNTD1 | Designates recombination intermediates for crossover formation |  | Mass spectrometry | (39) |
| CtIP | Promotes processing of DSBs to facilitate RPA loading | T527 | Mass spectrometry and experimental validation | (45,61) |
| CUL4A | Required for the repair of DSBs in spermatocytes | T276 | Mass spectrometry | (62) |
| EXO1 | Involved in resectioning of DSBs | S725, T732 | Mass spectrometry | (63) |
| GEN1 | Resolvase acitivty | S753, S757, S759, S794 | Mass spectrometry | (47) |
| HEI10 | Promotes RNF212 and/or MSH4-MSH5 turnover from chromosomes |  | Mass spectrometry | (52) |
| HFM1 | Required for normal numbers of crossovers |  | Mass spectrometry | (39) |
| MLH1 | Resolvase activity; cleaves DNA with Holiday junctions to achieve resolution and form class I crossovers | T363 | Mass spectrometry | (39,40,64) |
| MRE11 | Required for formation of ssDNA. Forms complex with RAD50 and NBS1. Required for the removal of the SPO11-oligo | S605, S667, S668 | Mass spectrometry and experimental validation | (46,65) |
| MSH4 | Crossover deignation | T830 | Mass spectrometry | (39) |
| NBS1 | Required for MRN complex location to DSB sites | S213 | Mass spectrometry | (46,47) |
| PCNA | Required for activation of MutL endonuceases | S10 | Mass spectrometry | (43,51) |
| RAD50 | Required for repair of meiotic DSBs | T198, S703, S717, S872, S900 | Mass spectrometry | (44,49) |
| RAD51B | Involved in homolog recognition | T66 | Mass spectrometry | (39) |
| RAD51C | Involved in homolog recognition | T60 | Mass spectrometry | (41) |
| RNF212 | Required for stabilizing crossover -fated intermediates |  | Mass spectrometry | (39) |
| RPA2 | Binds to ssDNA at DSB sites; prevents ssDNA from degrading |  | Mass spectrometry | (39,49) |
| SPO16 | Promotes formation of recombination intermediates and  crossovers. Required to stabilize the recombination  intermediates. | S4 | Mass spectrometry | (39) |
| SWSAP1 | Promotes RAD51- and DMC1-mediated stable presynaptic  filament formation |  | Mass spectrometry | (39) |
| TDP-43 | Required for homologous pairing and synapsis |  | Mass spectrometry and experimental validation | (46) |
| TEX15 | Required for loading of RAD51 and DMC1 at DSB sites in  males. | T1901, S2443 | Mass spectrometry | (39) |
| ZCWPW1 | Interacts with PRDM9 and recognizes H3K4me3 and  H3K36me3 to ensure efficient DSB repair | T68, T470 | Mass spectrometry | (39,44) |

***Table. S2 Antibody information***

| Antibody name | Manufacture (catalogue number) | Applications (working dilution) |
| --- | --- | --- |
| O-linked N-acetylglucosamine (O-GlcNAc) Monoclonal Antibody (RL2) | Invitrogen (MA1-072) | WB (1:1000)  IF (1:100) |
| OGT Polyclonal Antibody | Invitrogen (PA5-22071) | WB (1:1000) |
| MGEA5 Polyclonal antibody | Proteintech Biotechnology(14711-1-AP) | WB (1:1000)  IF (1:100) |
| Anti-MLH1 antibody | Abcam (ab91312) | IF (1:50) |
| Anti-Rec8 antibody | Abcam (ab192241) | IF (1:100) |
| Anti-Rpa2 antibody | Abcam (ab76420) | IF (1:100) |
| Anti-gamma H2AX (phospho S139) antibody | Abcam (ab81299) | IF (1:500) |
| Anti-SYCP3 Antibody | Abcam (ab97672) | IF (1:200) |
| Anti-Rad51 Antibody | Abcam (ab133534) | IF (1:50) |
| Anti-c-Kit Antibody | Abcam (ab32363) | IF (1:200) |
| Anti-Stra8 Antibody | Abcam (ab49602) | IF (1:400) |
| Anti-Lin28 Antibody | Abcam (ab63740) | IF (1:200) |
| FITC-labeled Goat Anti Rabbit Antibody | ZSGB bio (ZF-0311) | IF (1:50) |
| Alexa Fluor® 594 labeled Goat Anti Mouse Antibody | ZSGB bio (ZF-0513) | IF (1:50) |
| Goat anti-Mouse IgG (H+L) Secondary Antibody, HRP | Invitrogen (31430) | WB (1:5000) |
| Goat anti-Rabbit IgG (H+L) Secondary Antibody, HRP | Invitrogen (31460) | WB (1:5000) |

***Table S3 Information on the primers***

| Gene | Forward (5’-3’) | Reverse (5’-3’) |
| --- | --- | --- |
| *Gapdh* | TGACCTCAACTACATGGTCTACA | CTTCCCATTCTCGGCCTTG |
| *Hsd3b1* | TGGACAAAGTATTCCGACCAGA | GGCACACTTGCTTGAACACAG |
| *Star* | ATGTTCCTCGCTACGTTCAAG | CCCAGTGCTCTCCAGTTGAG |
| *Cldn11* | ATGGTAGCCACTTGCCTTCAG | AGTTCGTCCATTTTTCGGCAG |
| *Sox9* | AGTACCCGCATCTGCACAAC | ACGAAGGGTCTCTTCTCGCT |
| *Plzf* | CTGGGACTTTGTGCGATGTG | CGGTGGAAGAGGATCTCAAACA |
| *Lin28* | GGCATCTGTAAGTGGTTCAACG | GCCAGTGACACGGATGGATT |
| *C-kit* | GCCACGTCTCAGCCATCTG | GTCGCCAGCTTCAACTATTAACT |
| *Stra8* | ACAACCTAAGGAAGGCAGTTTAC | GACCTCCTCTAAGCTGTTGGG |
| *Sycp3* | AGACATGGGACATGAAGTAGGC | CCTCTCTCGTTCGTTCGTTTT |
| *Piwil1* | CAGCAACCTGGGTACATCCC | CCAAGGTCATGGAAGTCTCGG |
| *Acrv1* | TCAGCAACTTTCAAGCGAGTAT | CTCCTGAAGAGTGCTCACCTG |
| *Tex12* | TGGCAAACCACCTTGTAAAACC | TGCTCATATCGCTCAAATCCTTC |
| *Tnp1* | ACCAGCCGCAAGCTAAAGAC | TTTCCTACTTTTCAGGACGCTC |
| *Prm1* | TCCGACTCATGTTGAAAAACCC | CCTTCCCCGGATTCCTGTCT |
| *Rad51* | TGGCCAGATTTCTGAGGATG | CATGGCTGCTCCATCTACTT |
| *Dmc1* | AGATGTTGTCACGACTCCAAA | GGATCAGCCTGAAAGGTCATAG |

1. Johnson SL, Dunleavy J, Gemmell NJ, Nakagawa S. Consistent age-dependent declines in human semen quality: A systematic review and meta-analysis. Ageing Res Rev [Internet]. 2015;19:22–33. Available from: http://dx.doi.org/10.1016/j.arr.2014.10.007

2. Belloc F, Airiau K, Jeanneteau M, Garcia M, rin EG eacute, Lippert E, et al. Belloc_2009. 2009;23(4):679–85.

3. Klonoff-Cohen HS, Natarajan L. The effect of advancing paternal age on pregnancy and live birth rates in couples undergoing in vitro fertilization or gamete intrafallopian transfer. Am J Obstet Gynecol. 2004;191(2):507–14.

4. Li H, Chen H, Zhang X, Qi Y, Wang B, Cui Y, et al. Global phosphoproteomic analysis identified key kinases regulating male meiosis in mouse. Cell Mol Life Sci [Internet]. 2022; Available from: https://doi.org/10.1007/s00018-022-04507-8

5. Wang L, Cao C, Wang F, Zhao J, Li W. H2B ubiquitination: Conserved molecular mechanism, diverse physiologic functions of the E3 ligase during meiosis. Nucleus. 2017;8(5):461–8.

6. Yao C, Liu Y, Sun M, Niu M, Yuan Q, Hai Y, et al. MicroRNAs and DNA methylation as epigenetic regulators of mitosis, meiosis and spermiogenesis. Reproduction. 2015;150(1):R25–34.

7. Bhagwat N, Owens S, Ito M, Boinapalli J, Poa P, Ditzel A, et al. Sumo is a pervasive regulator of meiosis. Elife. 2021;10:1–89.

8. Perheentupa A, Huhtaniemi I. Aging of the human ovary and testis. Mol Cell Endocrinol. 2009;299(1):2–13.

9. Banerjee PS, Lagerlöf O, Hart GW. Roles of O-GlcNAc in chronic diseases of aging. Mol Aspects Med [Internet]. 2016;51:1–15. Available from: http://dx.doi.org/10.1016/j.mam.2016.05.005

10. Slawson C, Hart GW. O-GlcNAc signalling: Implications for cancer cell biology. Nat Rev Cancer. 2011 Sep;11(9):678–84.

11. Michalski J, Lefebvre T. Modulation of O-GlcNAc Glycosylation During Xenopus Oocyte Maturation. 2004;1010:999–1010.

12. Banerjee PS, Lagerlöf O, Hart GW. Roles of O-GlcNAc in chronic diseases of aging. Mol Aspects Med [Internet]. 2016;51:1–15. Available from: http://dx.doi.org/10.1016/j.mam.2016.05.005

13. Fayomi AP, Orwig KE. Spermatogonial stem cells and spermatogenesis in mice, monkeys and men. Stem Cell Res [Internet]. 2018;29(March):207–14. Available from: https://doi.org/10.1016/j.scr.2018.04.009

14. Lee R, Lee WY, Park HJ, Ha WT, Woo JS, Chung HJ, et al. Stage-specific expression of DDX4 and c-kit at different developmental stages of the porcine testis. Anim Reprod Sci. 2018;190(June 2017):18–26.

15. Koubova J, Hu YC, Bhattacharyya T, Soh YQS, Gill ME, Goodheart ML, et al. Retinoic Acid Activates Two Pathways Required for Meiosis in Mice. PLoS Genet. 2014;10(8).

16. Botelho RJ, DiNicolo L, Tsao N, Karaiskakis A, Tarsounas M, Moens PB, et al. The genomic structure of SYCP3, a meiosis-specific gene encoding a protein of the chromosome core. Biochim Biophys Acta - Gene Struct Expr. 2001;1518(3):294–9.

17. Zhang LF, Tan-Tai WJ, Li XH, Liu MF, Shi HJ, Martin-Deleon PA, et al. PHB regulates meiotic recombination via JAK2-mediated histone modifications in spermatogenesis. Nucleic Acids Res. 2020;48(9):4780–96.

18. Marcet-Ortega M, Pacheco S, Martínez-Marchal A, Castillo H, Flores E, Jasin M, et al. p53 and TAp63 participate in the recombination-dependent pachytene arrest in mouse spermatocytes. PLoS Genet. 2017;13(6):1–24.

19. Li T, Li X, Attri KS, Liu C, Li L, Herring LE, et al. O-GlcNAc Transferase Links Glucose Metabolism to MAVS-Mediated Antiviral Innate Immunity. Cell Host Microbe [Internet]. 2018;24(6):791-803.e6. Available from: https://doi.org/10.1016/j.chom.2018.11.001

20. Andrés-Bergós J, Tardio L, Larranaga-Vera A, Gómez R, Herrero-Beaumont G, Largo R. The increase in O-linked N-acetylglucosamine protein modification stimulates chondrogenic differentiation both in vitro and in vivo. J Biol Chem. 2012 Sep 28;287(40):33615–28.

21. Barchi M, Mahadevaiah S, Di Giacomo M, Baudat F, de Rooij DG, Burgoyne PS, et al. Surveillance of Different Recombination Defects in Mouse Spermatocytes Yields Distinct Responses despite Elimination at an Identical Developmental Stage. Mol Cell Biol. 2005;25(16):7203–15.

22. Hinch AG, Becker PW, Li T, Moralli D, Zhang G, Bycroft C, et al. The Configuration of RPA, RAD51, and DMC1 Binding in Meiosis Reveals the Nature of Critical Recombination Intermediates. Mol Cell [Internet]. 2020;79(4):689-701.e10. Available from: https://doi.org/10.1016/j.molcel.2020.06.015

23. Lee S-J, Sung R-J, Verdine GL. Mechanism of DNA Lesion Homing and Recognition by the Uvr Nucleotide Excision Repair System. Research. 2019;2019(Figure 1):1–11.

24. Zickler D, Kleckner N. of Homologs during Meiosis. Cold Spring Harb Lab Press [Internet]. 2015;1,2. Available from: http://cshperspectives.cshlp.org/

25. Chen L, Li Y, Song Z, Xue S, Liu F, Chang X, et al. O-GlcNAcylation promotes cerebellum development and medulloblastoma oncogenesis via SHH signaling. Proc Natl Acad Sci U S A. 2022;119(34):1–12.

26. Stone BA, Alex A, Werlin LB, Marrs RP. Age thresholds for changes in semen parameters in men. Fertil Steril [Internet]. 2013;100(4):952–8. Available from: http://dx.doi.org/10.1016/j.fertnstert.2013.05.046

27. Zitzmann M. Effects of age on male fertility. Best Pract Res Clin Endocrinol Metab [Internet]. 2013;27(4):617–28. Available from: http://dx.doi.org/10.1016/j.beem.2013.07.004

28. Johnson L, Nguyen HB, Petty CS, Neaves WB. Quantification of human spermatogenesis: germ cell degeneration during spermatocytogenesis and meiosis in testes from younger and older adult men. Biol Reprod. 1987;37(3):739–47.

29. Zhao H, Song L, Ma N, Liu C, Dun Y, Zhou Z, et al. The dynamic changes of Nrf2 mediated oxidative stress, DNA damage and base excision repair in testis of rats during aging. Exp Gerontol. 2021;152(June).

30. Sedelnikova OA, Horikawa I, Zimonjic DB, Popescu NC, Bonner WM, Barrett JC. Senescing human cells and ageing mice accumulate DNA lesions with unrepairable double-strand breaks. Nat Cell Biol. 2004;6(2):168–70.

31. Yang YR, Song M, Lee H, Jeon Y, Choi E, Jang H, et al. O-GlcNAcase is essential for embryonic development and maintenance of genomic stability Yong. Aging Cell. 2012;11:439–48.

32. Lin J, Xiang Y, Huang J, Zeng H, Zeng Y, Liu J, et al. NAT10 Maintains OGA mRNA Stability Through ac4C Modification in Regulating Oocyte Maturation. Front Endocrinol (Lausanne). 2022;13(July):1–11.

33. Keeney S, Giroux CN, Kleckner N. Meiosis-specific DNA double-strand breaks are catalyzed by Spo11, a member of a widely conserved protein family. Cell. 1997;88(3):375–84.

34. Fukuda T, Pratto F, Schimenti JC, Turner JMA, Camerini-Otero RD, Höög C. Phosphorylation of chromosome core components may serve as axis marks for the status of chromosomal events during mammalian meiosis. PLoS Genet. 2012;8(2).

35. Xie C, Wang W, Tu C, Meng L, Lu G, Lin G, et al. Meiotic recombination: insights into its mechanisms and its role in human reproduction with a special focus on non-obstructive azoospermia. Hum Reprod Update. 2022;1–35.

36. Chatham JC, Zhang J, Wende AR. Role of o-linked n-acetylglucosamine proteinmodification in cellular (Patho) physiology. Physiol Rev. 2021;101(2):427–93.

37. Wang S, Chen Q, Zhang Y, Zheng F, Xue T, Ge X, et al. Omega-3 polyunsaturated fatty acids alleviate hydrogen sulfide-induced blood-testis barrier disruption in the testes of adult mice. Reprod Toxicol [Internet]. 2020;98(July):233–41. Available from: https://doi.org/10.1016/j.reprotox.2020.10.007

38. Dia F, Strange T, Liang J, Hamilton J, Berkowitz KM. Preparation of meiotic chromosome spreads from mouse spermatocytes. J Vis Exp. 2017;2017(129):1–5.

39. Huo B, Zhang W, Zhao X, Dong H, Yu Y, Wang J, et al. A triarylphosphine-trimethylpiperidine reagent for the one-step derivatization and enrichment of protein post-translational modifications and identification by mass spectrometry. Chem Commun. 2018;54(98):13790–3.

40. Shen B, Zhang W, Shi Z, Tian F, Deng Y, Sun C, et al. A novel strategy for global mapping of O-GlcNAc proteins and peptides using selective enzymatic deglycosylation, HILIC enrichment and mass spectrometry identification. Talanta. 2017 Jul 1;169:195–202.

41. Qin W, Qin K, Fan X, Peng L, Hong W, Zhu Y, et al. Artificial Cysteine S-Glycosylation Induced by Per-O-Acetylated Unnatural Monosaccharides during Metabolic Glycan Labeling. Angew Chemie - Int Ed. 2018 Feb 12;57(7):1817–20.

42. Chen Q, Yu X. OGT restrains the expansion of DNA damage signaling. Nucleic Acids Res. 2016;44(19):9266–78.

43. Wong YK, Wang J, Lim TK, Lin Q, Yap CT, Shen HM. O-GlcNAcylation promotes fatty acid synthase activity under nutritional stress as a pro-survival mechanism in cancer cells. Proteomics. 2022 May 1;22(9).

44. Woo CM, Lund PJ, Huang AC, Davis MM, Bertozzi CR, Pitteri SJ. Mapping and quantification of over 2000 O-linked glycopeptides in activated human T cells with isotope-targeted glycoproteomics (Isotag). Mol Cell Proteomics. 2018 Apr 1;17(4):764–75.

45. Hahne H, Sobotzki N, Nyberg T, Helm D, Borodkin VS, Van Aalten DMF, et al. Proteome wide purification and identification of O-GlcNAc-modified proteins using click chemistry and mass spectrometry. J Proteome Res. 2013 Feb 1;12(2):927–36.

46. Xie X, Wu Q, Zhang K, Liu Y, Zhang N, Chen Q, et al. O-GlcNAc modification regulates MTA1 transcriptional activity during breast cancer cell genotoxic adaptation. Biochim Biophys Acta - Gen Subj. 2021 Aug 1;1865(8).

47. Liu J, Hao Y, Wang C, Jin Y, Yang Y, Gu J, et al. An Optimized Isotopic Photocleavable Tagging Strategy for Site-Specific and Quantitative Profiling of Protein O-GlcNAcylation in Colorectal Cancer Metastasis. ACS Chem Biol. 2022 Mar 18;17(3):513–20.

48. Chen Y, Tang F, Qin H, Yue X, Nie Y, Huang W, et al. Endo-M Mediated Chemoenzymatic Approach Enables Reversible Glycopeptide Labeling for O-GlcNAcylation Analysis. Angew Chemie - Int Ed. 2022 Jun 7;61(23).

49. Zhang W, Liu T, Dong H, Bai H, Tian F, Shi Z, et al. Synthesis of a Highly Azide-Reactive and Thermosensitive Biofunctional Reagent for Efficient Enrichment and Large-Scale Identification of O-GlcNAc Proteins by Mass Spectrometry. Anal Chem. 2017 Jun 6;89(11):5810–7.

50. Liu Y, Chen Q, Zhang N, Zhang K, Dou T, Cao Y, et al. Proteomic profiling and genome-wide mapping of O-GlcNAc chromatin-associated proteins reveal an O-GlcNAc-regulated genotoxic stress response. Nat Commun. 2020 Dec 1;11(1).

51. Lin CH, Liao CC, Wang SY, Peng CY, Yeh YC, Chen MY, et al. Comparative O-GlcNAc Proteomic Analysis Reveals a Role of O-GlcNAcylated SAM68 in Lung Cancer Aggressiveness. Cancers (Basel). 2022 Jan 1;14(1).

52. Levine ZG, Fan C, Melicher MS, Orman M, Benjamin T, Walker S. O -GlcNAc Transferase Recognizes Protein Substrates Using an Asparagine Ladder in the Tetratricopeptide Repeat (TPR) Superhelix. J Am Chem Soc. 2018 Mar 14;140(10):3510–3.

53. Phoomak C, Park D, Silsirivanit A, Sawanyawisuth K, Vaeteewoottacharn K, Detarya M, et al. O-GlcNAc-induced nuclear translocation of hnRNP-K is associated with progression and metastasis of cholangiocarcinoma. Mol Oncol. 2019 Feb 1;13(2):338–57.

54. Song J, Liu C, Wang X, Xu B, Liu X, Li Y, et al. O-GlcNAcylation Quantification of Certain Protein by the Proximity Ligation Assay and Clostridium perfringen OGAD298N(CpOGAD298N). ACS Chem Biol. 2021 Jun 18;16(6):1040–9.

55. Shu XE, Mao Y, Jia L, Qian SB. Dynamic eIF3a O-GlcNAcylation controls translation reinitiation during nutrient stress. Nat Chem Biol. 2022 Feb 1;18(2):134–41.

56. Qin K, Zhu Y, Qin W, Gao J, Shao X, Wang YL, et al. Quantitative Profiling of Protein O-GlcNAcylation Sites by an Isotope-Tagged Cleavable Linker. ACS Chem Biol. 2018 Aug 17;13(8):1983–9.

57. Chen Q, Yu X. OGT restrains the expansion of DNA damage signaling. Nucleic Acids Res. 2016 Nov 2;44(19):9266–78.

58. Chattopadhyay T, Maniyadath B, Bagul HP, Chakraborty A, Shukla N, Budnar S, et al. Spatiotemporal gating of SIRT1 functions by O-GlcNAcylation is essential for liver metabolic switching and prevents hyperglycemia. Proc Natl Acad Sci U S A. 2020 Mar 24;117(12):6890–900.

59. Shan H, Sun J, Shi M, Liu X, Shi Z, Yu W, et al. Generation and characterization of a site-specific antibody for SIRT1 O-GlcNAcylated at serine 549. Glycobiology. 2018 Jul 1;28(7):482–7.

60. Han C, Gu Y, Shan H, Mi W, Sun J, Shi M, et al. O-GlcNAcylation of SIRT1 enhances its deacetylase activity and promotes cytoprotection under stress. Nat Commun. 2017 Dec 1;8(1).

61. Hao Y, Fan X, Shi Y, Zhang C, Sun D en, Qin K, et al. Next-generation unnatural monosaccharides reveal that ESRRB O-GlcNAcylation regulates pluripotency of mouse embryonic stem cells. Nat Commun. 2019 Dec 1;10(1).

62. Ramirez DH, Yang B, D’Souza AK, Shen D, Woo CM. Truncation of the TPR domain of OGT alters substrate and glycosite selection. Anal Bioanal Chem. 2021 Dec 1;413(30):7385–99.

63. Li J, Li Z, Duan X, Qin K, Dang L, Sun S, et al. An Isotope-Coded Photocleavable Probe for Quantitative Profiling of Protein O-GlcNAcylation. ACS Chem Biol. 2019 Jan 18;14(1):4–10.

64. Xu S, Sun F, Wu R. A Chemoenzymatic Method Based on Easily Accessible Enzymes for Profiling Protein O-GlcNAcylation. Anal Chem. 2020 Jul 21;92(14):9807–14.

65. Gondane A, Girmay S, Helevä A, Pallasaho S, Loda M, Itkonen HM. O-GlcNAc transferase couples MRE11 to transcriptionally active chromatin to suppress DNA damage. J Biomed Sci. 2022 Dec 1;29(1).

1. Johnson SL, Dunleavy J, Gemmell NJ, Nakagawa S. Consistent age-dependent declines in human semen quality: A systematic review and meta-analysis. Ageing Res Rev [Internet]. 2015;19:22–33. Available from: http://dx.doi.org/10.1016/j.arr.2014.10.007

2. Belloc F, Airiau K, Jeanneteau M, Garcia M, rin EG eacute, Lippert E, et al. Belloc_2009. 2009;23(4):679–85.

3. Klonoff-Cohen HS, Natarajan L. The effect of advancing paternal age on pregnancy and live birth rates in couples undergoing in vitro fertilization or gamete intrafallopian transfer. Am J Obstet Gynecol. 2004;191(2):507–14.

4. Li H, Chen H, Zhang X, Qi Y, Wang B, Cui Y, et al. Global phosphoproteomic analysis identified key kinases regulating male meiosis in mouse. Cell Mol Life Sci [Internet]. 2022; Available from: https://doi.org/10.1007/s00018-022-04507-8

5. Wang L, Cao C, Wang F, Zhao J, Li W. H2B ubiquitination: Conserved molecular mechanism, diverse physiologic functions of the E3 ligase during meiosis. Nucleus. 2017;8(5):461–8.

6. Yao C, Liu Y, Sun M, Niu M, Yuan Q, Hai Y, et al. MicroRNAs and DNA methylation as epigenetic regulators of mitosis, meiosis and spermiogenesis. Reproduction. 2015;150(1):R25–34.

7. Bhagwat N, Owens S, Ito M, Boinapalli J, Poa P, Ditzel A, et al. Sumo is a pervasive regulator of meiosis. Elife. 2021;10:1–89.

8. Perheentupa A, Huhtaniemi I. Aging of the human ovary and testis. Mol Cell Endocrinol. 2009;299(1):2–13.

9. Banerjee PS, Lagerlöf O, Hart GW. Roles of O-GlcNAc in chronic diseases of aging. Mol Aspects Med [Internet]. 2016;51:1–15. Available from: http://dx.doi.org/10.1016/j.mam.2016.05.005

10. Slawson C, Hart GW. O-GlcNAc signalling: Implications for cancer cell biology. Nat Rev Cancer. 2011 Sep;11(9):678–84.

11. Michalski J, Lefebvre T. Modulation of O-GlcNAc Glycosylation During Xenopus Oocyte Maturation. 2004;1010:999–1010.

12. Banerjee PS, Lagerlöf O, Hart GW. Roles of O-GlcNAc in chronic diseases of aging. Mol Aspects Med [Internet]. 2016;51:1–15. Available from: http://dx.doi.org/10.1016/j.mam.2016.05.005

13. Fayomi AP, Orwig KE. Spermatogonial stem cells and spermatogenesis in mice, monkeys and men. Stem Cell Res [Internet]. 2018;29(March):207–14. Available from: https://doi.org/10.1016/j.scr.2018.04.009

14. Lee R, Lee WY, Park HJ, Ha WT, Woo JS, Chung HJ, et al. Stage-specific expression of DDX4 and c-kit at different developmental stages of the porcine testis. Anim Reprod Sci. 2018;190(June 2017):18–26.

15. Koubova J, Hu YC, Bhattacharyya T, Soh YQS, Gill ME, Goodheart ML, et al. Retinoic Acid Activates Two Pathways Required for Meiosis in Mice. PLoS Genet. 2014;10(8).

16. Botelho RJ, DiNicolo L, Tsao N, Karaiskakis A, Tarsounas M, Moens PB, et al. The genomic structure of SYCP3, a meiosis-specific gene encoding a protein of the chromosome core. Biochim Biophys Acta - Gene Struct Expr. 2001;1518(3):294–9.

17. Zhang LF, Tan-Tai WJ, Li XH, Liu MF, Shi HJ, Martin-Deleon PA, et al. PHB regulates meiotic recombination via JAK2-mediated histone modifications in spermatogenesis. Nucleic Acids Res. 2020;48(9):4780–96.

18. Marcet-Ortega M, Pacheco S, Martínez-Marchal A, Castillo H, Flores E, Jasin M, et al. p53 and TAp63 participate in the recombination-dependent pachytene arrest in mouse spermatocytes. PLoS Genet. 2017;13(6):1–24.

19. Li T, Li X, Attri KS, Liu C, Li L, Herring LE, et al. O-GlcNAc Transferase Links Glucose Metabolism to MAVS-Mediated Antiviral Innate Immunity. Cell Host Microbe [Internet]. 2018;24(6):791-803.e6. Available from: https://doi.org/10.1016/j.chom.2018.11.001

20. Andrés-Bergós J, Tardio L, Larranaga-Vera A, Gómez R, Herrero-Beaumont G, Largo R. The increase in O-linked N-acetylglucosamine protein modification stimulates chondrogenic differentiation both in vitro and in vivo. J Biol Chem. 2012 Sep 28;287(40):33615–28.

21. Barchi M, Mahadevaiah S, Di Giacomo M, Baudat F, de Rooij DG, Burgoyne PS, et al. Surveillance of Different Recombination Defects in Mouse Spermatocytes Yields Distinct Responses despite Elimination at an Identical Developmental Stage. Mol Cell Biol. 2005;25(16):7203–15.

22. Hinch AG, Becker PW, Li T, Moralli D, Zhang G, Bycroft C, et al. The Configuration of RPA, RAD51, and DMC1 Binding in Meiosis Reveals the Nature of Critical Recombination Intermediates. Mol Cell [Internet]. 2020;79(4):689-701.e10. Available from: https://doi.org/10.1016/j.molcel.2020.06.015

23. Lee S-J, Sung R-J, Verdine GL. Mechanism of DNA Lesion Homing and Recognition by the Uvr Nucleotide Excision Repair System. Research. 2019;2019(Figure 1):1–11.

24. Zickler D, Kleckner N. of Homologs during Meiosis. Cold Spring Harb Lab Press [Internet]. 2015;1,2. Available from: http://cshperspectives.cshlp.org/

25. Chen L, Li Y, Song Z, Xue S, Liu F, Chang X, et al. O-GlcNAcylation promotes cerebellum development and medulloblastoma oncogenesis via SHH signaling. Proc Natl Acad Sci U S A. 2022;119(34):1–12.

26. Stone BA, Alex A, Werlin LB, Marrs RP. Age thresholds for changes in semen parameters in men. Fertil Steril [Internet]. 2013;100(4):952–8. Available from: http://dx.doi.org/10.1016/j.fertnstert.2013.05.046

27. Zitzmann M. Effects of age on male fertility. Best Pract Res Clin Endocrinol Metab [Internet]. 2013;27(4):617–28. Available from: http://dx.doi.org/10.1016/j.beem.2013.07.004

28. Johnson L, Nguyen HB, Petty CS, Neaves WB. Quantification of human spermatogenesis: germ cell degeneration during spermatocytogenesis and meiosis in testes from younger and older adult men. Biol Reprod. 1987;37(3):739–47.

29. Zhao H, Song L, Ma N, Liu C, Dun Y, Zhou Z, et al. The dynamic changes of Nrf2 mediated oxidative stress, DNA damage and base excision repair in testis of rats during aging. Exp Gerontol. 2021;152(June).

30. Sedelnikova OA, Horikawa I, Zimonjic DB, Popescu NC, Bonner WM, Barrett JC. Senescing human cells and ageing mice accumulate DNA lesions with unrepairable double-strand breaks. Nat Cell Biol. 2004;6(2):168–70.

31. Yang YR, Song M, Lee H, Jeon Y, Choi E, Jang H, et al. O-GlcNAcase is essential for embryonic development and maintenance of genomic stability Yong. Aging Cell. 2012;11:439–48.

32. Lin J, Xiang Y, Huang J, Zeng H, Zeng Y, Liu J, et al. NAT10 Maintains OGA mRNA Stability Through ac4C Modification in Regulating Oocyte Maturation. Front Endocrinol (Lausanne). 2022;13(July):1–11.

33. Keeney S, Giroux CN, Kleckner N. Meiosis-specific DNA double-strand breaks are catalyzed by Spo11, a member of a widely conserved protein family. Cell. 1997;88(3):375–84.

34. Fukuda T, Pratto F, Schimenti JC, Turner JMA, Camerini-Otero RD, Höög C. Phosphorylation of chromosome core components may serve as axis marks for the status of chromosomal events during mammalian meiosis. PLoS Genet. 2012;8(2).

35. Xie C, Wang W, Tu C, Meng L, Lu G, Lin G, et al. Meiotic recombination: insights into its mechanisms and its role in human reproduction with a special focus on non-obstructive azoospermia. Hum Reprod Update. 2022;1–35.

36. Chatham JC, Zhang J, Wende AR. Role of o-linked n-acetylglucosamine proteinmodification in cellular (Patho) physiology. Physiol Rev. 2021;101(2):427–93.

37. Wang S, Chen Q, Zhang Y, Zheng F, Xue T, Ge X, et al. Omega-3 polyunsaturated fatty acids alleviate hydrogen sulfide-induced blood-testis barrier disruption in the testes of adult mice. Reprod Toxicol [Internet]. 2020;98(July):233–41. Available from: https://doi.org/10.1016/j.reprotox.2020.10.007

38. Dia F, Strange T, Liang J, Hamilton J, Berkowitz KM. Preparation of meiotic chromosome spreads from mouse spermatocytes. J Vis Exp. 2017;2017(129):1–5.

39. Huo B, Zhang W, Zhao X, Dong H, Yu Y, Wang J, et al. A triarylphosphine-trimethylpiperidine reagent for the one-step derivatization and enrichment of protein post-translational modifications and identification by mass spectrometry. Chem Commun. 2018;54(98):13790–3.

40. Shen B, Zhang W, Shi Z, Tian F, Deng Y, Sun C, et al. A novel strategy for global mapping of O-GlcNAc proteins and peptides using selective enzymatic deglycosylation, HILIC enrichment and mass spectrometry identification. Talanta. 2017 Jul 1;169:195–202.

41. Qin W, Qin K, Fan X, Peng L, Hong W, Zhu Y, et al. Artificial Cysteine S-Glycosylation Induced by Per-O-Acetylated Unnatural Monosaccharides during Metabolic Glycan Labeling. Angew Chemie - Int Ed. 2018 Feb 12;57(7):1817–20.

42. Chen Q, Yu X. OGT restrains the expansion of DNA damage signaling. Nucleic Acids Res. 2016;44(19):9266–78.

43. Wong YK, Wang J, Lim TK, Lin Q, Yap CT, Shen HM. O-GlcNAcylation promotes fatty acid synthase activity under nutritional stress as a pro-survival mechanism in cancer cells. Proteomics. 2022 May 1;22(9).

44. Woo CM, Lund PJ, Huang AC, Davis MM, Bertozzi CR, Pitteri SJ. Mapping and quantification of over 2000 O-linked glycopeptides in activated human T cells with isotope-targeted glycoproteomics (Isotag). Mol Cell Proteomics. 2018 Apr 1;17(4):764–75.

45. Hahne H, Sobotzki N, Nyberg T, Helm D, Borodkin VS, Van Aalten DMF, et al. Proteome wide purification and identification of O-GlcNAc-modified proteins using click chemistry and mass spectrometry. J Proteome Res. 2013 Feb 1;12(2):927–36.

46. Xie X, Wu Q, Zhang K, Liu Y, Zhang N, Chen Q, et al. O-GlcNAc modification regulates MTA1 transcriptional activity during breast cancer cell genotoxic adaptation. Biochim Biophys Acta - Gen Subj. 2021 Aug 1;1865(8).

47. Liu J, Hao Y, Wang C, Jin Y, Yang Y, Gu J, et al. An Optimized Isotopic Photocleavable Tagging Strategy for Site-Specific and Quantitative Profiling of Protein O-GlcNAcylation in Colorectal Cancer Metastasis. ACS Chem Biol. 2022 Mar 18;17(3):513–20.

48. Chen Y, Tang F, Qin H, Yue X, Nie Y, Huang W, et al. Endo-M Mediated Chemoenzymatic Approach Enables Reversible Glycopeptide Labeling for O-GlcNAcylation Analysis. Angew Chemie - Int Ed. 2022 Jun 7;61(23).

49. Zhang W, Liu T, Dong H, Bai H, Tian F, Shi Z, et al. Synthesis of a Highly Azide-Reactive and Thermosensitive Biofunctional Reagent for Efficient Enrichment and Large-Scale Identification of O-GlcNAc Proteins by Mass Spectrometry. Anal Chem. 2017 Jun 6;89(11):5810–7.

50. Liu Y, Chen Q, Zhang N, Zhang K, Dou T, Cao Y, et al. Proteomic profiling and genome-wide mapping of O-GlcNAc chromatin-associated proteins reveal an O-GlcNAc-regulated genotoxic stress response. Nat Commun. 2020 Dec 1;11(1).

51. Lin CH, Liao CC, Wang SY, Peng CY, Yeh YC, Chen MY, et al. Comparative O-GlcNAc Proteomic Analysis Reveals a Role of O-GlcNAcylated SAM68 in Lung Cancer Aggressiveness. Cancers (Basel). 2022 Jan 1;14(1).

52. Levine ZG, Fan C, Melicher MS, Orman M, Benjamin T, Walker S. O -GlcNAc Transferase Recognizes Protein Substrates Using an Asparagine Ladder in the Tetratricopeptide Repeat (TPR) Superhelix. J Am Chem Soc. 2018 Mar 14;140(10):3510–3.

53. Phoomak C, Park D, Silsirivanit A, Sawanyawisuth K, Vaeteewoottacharn K, Detarya M, et al. O-GlcNAc-induced nuclear translocation of hnRNP-K is associated with progression and metastasis of cholangiocarcinoma. Mol Oncol. 2019 Feb 1;13(2):338–57.

54. Song J, Liu C, Wang X, Xu B, Liu X, Li Y, et al. O-GlcNAcylation Quantification of Certain Protein by the Proximity Ligation Assay and Clostridium perfringen OGAD298N(CpOGAD298N). ACS Chem Biol. 2021 Jun 18;16(6):1040–9.

55. Shu XE, Mao Y, Jia L, Qian SB. Dynamic eIF3a O-GlcNAcylation controls translation reinitiation during nutrient stress. Nat Chem Biol. 2022 Feb 1;18(2):134–41.

56. Qin K, Zhu Y, Qin W, Gao J, Shao X, Wang YL, et al. Quantitative Profiling of Protein O-GlcNAcylation Sites by an Isotope-Tagged Cleavable Linker. ACS Chem Biol. 2018 Aug 17;13(8):1983–9.

57. Chen Q, Yu X. OGT restrains the expansion of DNA damage signaling. Nucleic Acids Res. 2016 Nov 2;44(19):9266–78.

58. Chattopadhyay T, Maniyadath B, Bagul HP, Chakraborty A, Shukla N, Budnar S, et al. Spatiotemporal gating of SIRT1 functions by O-GlcNAcylation is essential for liver metabolic switching and prevents hyperglycemia. Proc Natl Acad Sci U S A. 2020 Mar 24;117(12):6890–900.

59. Shan H, Sun J, Shi M, Liu X, Shi Z, Yu W, et al. Generation and characterization of a site-specific antibody for SIRT1 O-GlcNAcylated at serine 549. Glycobiology. 2018 Jul 1;28(7):482–7.

60. Han C, Gu Y, Shan H, Mi W, Sun J, Shi M, et al. O-GlcNAcylation of SIRT1 enhances its deacetylase activity and promotes cytoprotection under stress. Nat Commun. 2017 Dec 1;8(1).

61. Hao Y, Fan X, Shi Y, Zhang C, Sun D en, Qin K, et al. Next-generation unnatural monosaccharides reveal that ESRRB O-GlcNAcylation regulates pluripotency of mouse embryonic stem cells. Nat Commun. 2019 Dec 1;10(1).

62. Ramirez DH, Yang B, D’Souza AK, Shen D, Woo CM. Truncation of the TPR domain of OGT alters substrate and glycosite selection. Anal Bioanal Chem. 2021 Dec 1;413(30):7385–99.

63. Li J, Li Z, Duan X, Qin K, Dang L, Sun S, et al. An Isotope-Coded Photocleavable Probe for Quantitative Profiling of Protein O-GlcNAcylation. ACS Chem Biol. 2019 Jan 18;14(1):4–10.

64. Xu S, Sun F, Wu R. A Chemoenzymatic Method Based on Easily Accessible Enzymes for Profiling Protein O-GlcNAcylation. Anal Chem. 2020 Jul 21;92(14):9807–14.

65. Gondane A, Girmay S, Helevä A, Pallasaho S, Loda M, Itkonen HM. O-GlcNAc transferase couples MRE11 to transcriptionally active chromatin to suppress DNA damage. J Biomed Sci. 2022 Dec 1;29(1).
