# Supplementary material for: Age-related elevation of O-GlcNAc causes meiotic arrest in male mice
Source: Cell Death Discov. 2023 May 15;9:163. doi: 10.1038/s41420-023-01433-x (PMC10185674; doi:10.1038/s41420-023-01433-x)
Supplement: Supplementary file 11 — Supplementary Figure Legend [file 41420_2023_1433_MOESM11_ESM.docx]

***Figure. S1 Aged male mice show decreased fertility.*** *(A) H&E-stained sections of seminiferous tubules from 2-month and 16-month-old mice. (B) Measurement of the thickness of the seminiferous epithelium from 2-month and 16-month-old mice. (C) Testis organ index of 2-month-old and 16-month-old mice. (D-F) The concentration, progressive motility and total motility of sperm collected from the caudal epididymis of 2-month-old and 16-month-old mice using CASA assays. N=3. *p < 0.05, ** p <0.01, *** p <0.001*

***Figure. S2 Meiotic progression is impaired in aged mice.*** *(A) Immunostaining of SYCP3 (green) and γH2AX (red) in spermatocytes from 2-month-old mice (left) and 16-month-old mice (right).* *Scare bar=5μm. (B) The rate of abnormal γH2AX staining in pachytene spermatocytes in 2-month mice and 16-month mice. (C) The proportion of meiotic stages in testes of 2-month-old mice and 16-month-old mice. 200 spermatocytes were evaluated in each testis. The proportion of each substage of spermatocyte is calculated by dividing the number of such cells by the total number of spermatocytes. N=3. *p < 0.05, ** p <0.01, *** p <0.001.*

***Figure S3***  ***OGA decreases and O-GlcNAc elevates in testes of aged mice.*** *(A) Representative immunofluorescence images of testicular slices stained with OGT. (B)Fluorescence intensities of O-GlcNAc and OGA from Figure 3 quantified by ImageJ.*

***Figure. S4 Thiamet-G treatment elevates O-GlcNAcylation in testes.*** *(A) Western blot images of O-GlcNAc in the testes of mice that were treated with 0, 10, 20, 30, or 80 mg/kg Thiamet-G once. (B) Western blot image of O-GlcNAc in the testes of mice treated with PBS and 30 mg/kg Thiamet-G for 35 days. GAPDH was used as the internal reference.*

***Figure. S5 Thiamet-G treatment does not influence hormones in mice.*** *(A-D) The levels of testosterone, FSH, LH and inhibin B in the serum of control group and TMG group mice. N=5.*

***Figure S6 Elevation of O-GlcNAc impairs spermatogenesis.*** *(A) Upper panel: schematic showing the morphology of spermatogonia, spermatocytes and round spermatids. Lower panel: proportions of different cell types in the control group and TMG group.* *50 tubules were evaluated in each testis.* *The proportion of each type of cell is calculated by dividing the number of such cells by the total number of spermatogonia, spermatocytes, and round spermatid. (B) mRNA levels of spermatogenesis-related genes in the control group and TMG group as measured by qRT‒PCR. Gapdh was used as the internal reference. Means ± SD. N=5. *p < 0.05, ** p <0.01, *** p <0.001.*

***Figure. S7 Thiamet-G treatment induces apoptosis in the testes of mice.*** *(A) TUNEL staining images of testes slices from control and TMG group mice. The arrows represent apoptotic cells. (B) Positive TUNEL cells per seminiferous tubule in control and TMG group mice. (C) The rate of tubules with at least one apoptotic cell in control and TMG group mice. N=5. *p < 0.05, ** p <0.01, *** p <0.001*

***Figure.S8 The effects of OSMI-1 treatment on expressions of genes associated with spermatogenesis.*** *Gapdh was used as the internal reference. N=3. Means ± SD. *p < 0.05, ** p <0.01, *** p <0.001.*
