# Supplementary material for: Age-related elevation of O-GlcNAc causes meiotic arrest in male mice
Source: Cell Death Discov. 2023 May 15;9:163. doi: 10.1038/s41420-023-01433-x (PMC10185674; doi:10.1038/s41420-023-01433-x)
Supplement: Supplementary file 12 — Original Data File [file 41420_2023_1433_MOESM12_ESM.docx]

**Figure 2**





oga





RL2





OGT





GAPDH

**Figure 7**





O-GlcNAc


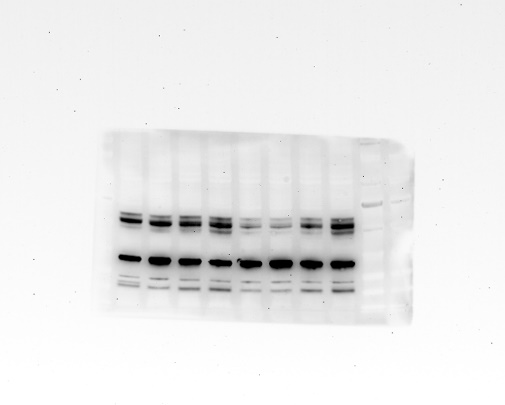


GAPDH





IP: O-GlcNAc WB: REC8





REC8





GAPDH

**Figure S4**


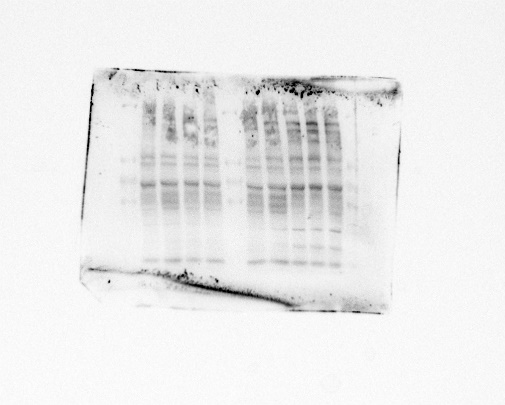


O-GlcNAc


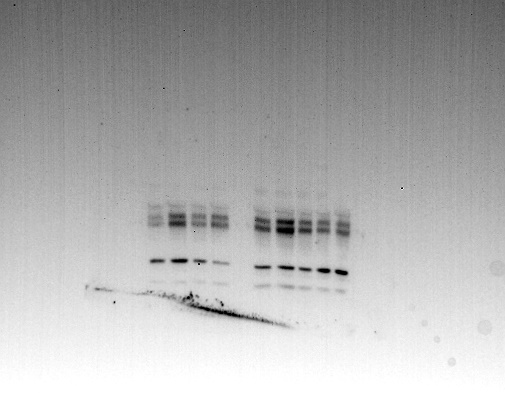


GAPDH


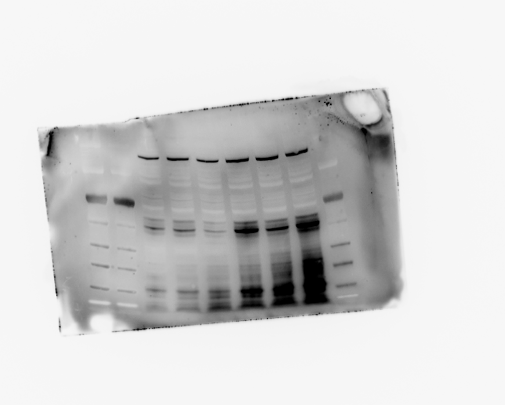


O-GlcNAc





GAPDH
