# Supplementary material for: Age-related elevation of O-GlcNAc causes meiotic arrest in male mice
Source: Cell Death Discov. 2023 May 15;9:163. doi: 10.1038/s41420-023-01433-x (PMC10185674; doi:10.1038/s41420-023-01433-x)
Supplement: Supplementary file 1 — Change of authorship [file 41420_2023_1433_MOESM1_ESM.docx]

Dear editor

During the revision of this manuscript, Dr Yang Yang provided help for the experiments. Therefore, we plan to add Dr. Yang Yang as our co-author. We have emailed this change to all the co-authors and collected these replies as follows. Thank you very much.
